# Downregulation of microRNA-27b-3p enhances tamoxifen resistance in breast cancer by increasing NR5A2 and CREB1 expression

**DOI:** 10.1038/cddis.2016.361

**Published:** 2016-11-03

**Authors:** Jiang Zhu, Zhengzhi Zou, Peipei Nie, Xiaoni Kou, Baoyan Wu, Songmao Wang, Zhangjun Song, Jianjun He

**Affiliations:** 1 Department of Breast Surgery, The First Affiliated Hospital, Xi'an Jiaotong University, Xi'an, Shaanxi 710061, China; 2 Department of Breast Disease, The Tumour Hospital of Shaanxi Province, Xi'an, Shaanxi 710061, China; 3 MOE Key Laboratory of Laser Life Science and Institute of Laser Life Science, College of Biophotonics, South China Normal University, Guangzhou, Guangdong 510631, China; 4 KingMed Diagnostics and KingMed School of Laboratory Medicine, Guangzhou Medical University, Guangzhou, Guangdong 510631, China; 5 Department of Hepatopathy, First Affiliated Hospital of Shaanxi Chinese Medicine University, Xianyang, Shaanxi 712000, China

## Abstract

Estrogen-dependent breast cancer is often treated with the aromatase inhibitors or estrogen receptor (ER) antagonists. Tamoxifen as a major ER antagonist is usually used to treat those patients with ER*α*-positive breast cancer. However, a majority of patients with ER*α* positive fail to respond to tamoxifen due to the presence of intrinsic or acquired resistance to the drug. Altered expression and functions of microRNAs (miRNAs) have been reportedly associated with tamoxifen resistance. In this study, we investigated the role of miR-27b-3p in resistance of breast cancer to tamoxifen. MiR-27b-3p levels were remarkably reduced in the tamoxifen-resistant breast cancer cells compared with their parental cells. In addition, miR-27b-3p was also significantly downregulated in breast tumor tissues relative to adjacent non-tumor tissues. Moreover, the expression levels of miR-27b-3p were lower in the breast cancer tissues from tamoxifen-resistant patients compared with that from untreated-tamoxifen patients. Notably, tamoxifen repressed miR-27b-3p expression, whereas estrogen induced miR-27b-3p expression in breast cancer cells. Besides, we provided experimental evidences that miR-27b-3p enhances the sensitivity of breast cancer cells to tamoxifen *in vitro* and *in vivo* models. More importantly, we validated that miR-27b-3p directly targeted and inhibited the expression of nuclear receptor subfamily 5 group A member 2 (NR5A2) and cAMP-response element binding protein 1 (CREB1) and therefore augmented tamoxifen-induced cytotoxicity in breast cancer. Lastly, miR-27b-3p levels were found to be significantly negatively correlated with both NR5A2 and CREB1 levels in breast cancer tissues. Our findings provided further evidence that miR-27b-3p might be considered as a novel and potential target for the diagnosis and treatment of tamoxifen-resistant breast cancer.

Over two-thirds of breast cancers overexpress estrogen receptor (ER), which contributes to breast cancer tumorigenesis and progression.^1^ Targeted inhibition of ER using selective modulators is considered the optimal treatment for breast cancer patients with ER-positive tumors. The selective ER modulator tamoxifen is an effective first-line endocrine therapy drug. It is found clinically that tamoxifen can significantly improve overall and relapse-free survival rates of all stages of patients with ER-positive breast cancer.^2^ Studies showed tamoxifen can reduce the incidence of contralateral breast cancer.^3^ Therefore, the drug has been approved as a prophylactic agent to prevent breast tumor.^3^ Despite their documented benefits in the management of patients with potentially endocrine-responsive breast cancers, an intrinsic or acquired resistance to tamoxifen is common in a significant proportion of those patients treated with the drug.^4, 5^

MicroRNAs (miRNAs) are a class of small non-coding RNAs that post-transcriptionally block expression of the target genes by directly interacting with mRNA of the genes. Numerous miRNAs have been discovered in human cells; however, their targets and function remain largely unknown. Dysregulated miRNA expression is frequently involved in the development of many human tumor types.^6, 7^ In addition, studies show involvement of miRNA in resistance of cancer cells to chemotherapeutic drugs.^8, 9, 10^ Moreover, upregulation of miRNAs expression causes tamoxifen resistance in breast cancer cells, such as miR-221/222,^11^ whereas miR-342 and miR-378a confer tamoxifen sensitivity by inhibiting their target genes.^12, 13^

MiRNA-27b-3p is one of the few miRNAs differentially expressed between tamoxifen-sensitive and -resistant breast cancer cell lines.^14^ It has reported that marked downregulation of miRNA-27b-3p in tamoxifen-resistant cells compared with parental MCF-7 cells.^14^ Recently, several studies have identified miR-27b-3p (also known as miR-27b) promotes cell proliferation and invasion in glioma and breast cancer,^15, 16^ and blocks paclitaxel-induced apoptosis in cervical cancer.^17^ MiR-27b-3p also reportedly plays a cancer-promoting role and is associated with poor prognosis in triple-negative breast cancer patients.^17^ On the other hand, miR-27b-3p functions as a tumor suppressor to inhibit cells growth, tumor progression and the inflammatory response by inhibiting the expression of PPARγ in neuroblastoma.^18^ Moreover, miR-27b-3p attenuates the acquisition of cancer stem cell properties in luminal-type breast cancer by repression of ENPP1.^19^ These findings suggest that the functions of miR-27b-3p are diverse and may be dependent on the specific cancer types.

In the present study, we developed a tamoxifen-resistant breast cancer cell model and investigated the potential roles of miR-27b-3p in the acquisition of tamoxifen resistance. We found that miR-27b-3p expressions were remarkably reduced in tamoxifen-resistant cells compared with their parental cells. In addition, miR-27b-3p was also significantly downregulated in the breast tumor tissues relative to their adjacent non-tumor tissues. Moreover, the expression levels of miR-27b-3p were lower in the breast cancer tissues from tamoxifen-resistant patients compared with that from untreated-tamoxifen patients. Additionally, we provided experimental evidences that miR-27b-3p enhances sensitivity of breast cancer cells to tamoxifen *in vitro* and *in vivo* models. Furthermore, we validated that miR-27b-3p directly targeted and inhibited the expression of nuclear receptor subfamily 5 group A member 2 (NR5A2) and cAMP-response element binding protein 1 (CREB1) and therefore augmented tamoxifen-induced cytotoxicity in breast cancer. Lastly, miR-27b-3p levels were found to be significantly negatively correlated with both NR5A2 and CREB1 levels in breast cancer tissues.

## Results

### Downregulation of miR-27b-3p in tamoxifen-resistant breast cancer cells and tissues

**Figure 1:**
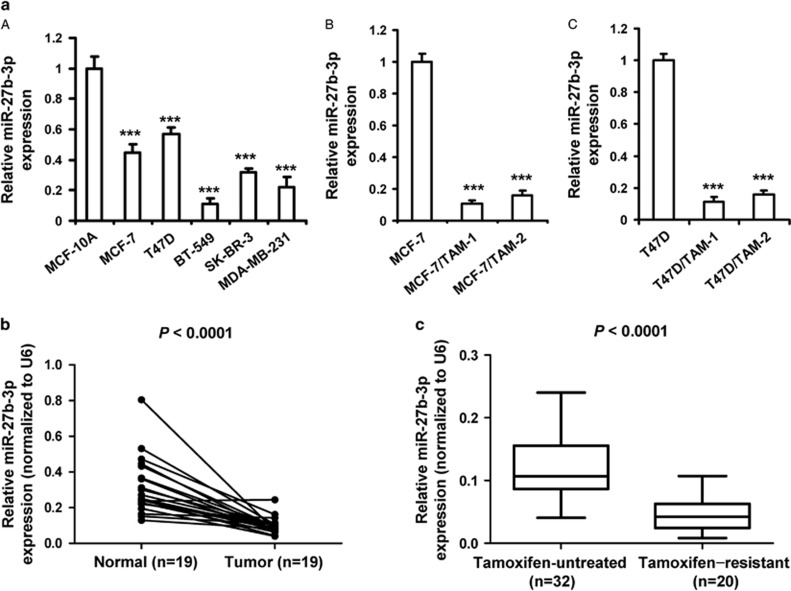
Downregulation of miR-27b-3p in tamoxifen-resistant breast cancer. (**a**) (**A**) MiR-27b-3p expression in different breast cell lines as measured by RT-PCR analysis and relative to their expression levels in MCF-10A cells. (**B** and **C**) MiR-27b-3p expression in tamoxifen-resistant MCF-7 and T47D cells as measured by RT-PCR analysis and relative to their expression levels in parental MCF-7 and T47D cells. The experiments were repeated three times. Data represent mean±S.D. ***P<0.001. (**b**) The expression levels of miR-27b-3p in tumor tissues compared with adjacent normal tissues in 19 breast cancer patients, *P*<0.001 by paired *t*-test. (**c**) The expression levels of miR-27b-3p in 32 tamoxifen untreated breast cancer tissues and 20 tamoxifen-resistant breast cancer tissues

To evaluate the expression of miR-27b-3p in breast cell lines, RT-PCR was performed in the five breast cancer cell lines, including MCF-7, T47D, BT-549, SK-BR-3 and MDA-MB-231 cells, and a noncancerous breast epithelial cell line MCF-10A cells. We found miR-27b-3p was significantly downregulated in the five cancer cell lines compared with MCF-10A. Notably, the levels of miR-27b-3p in the two ER-positive (ER-positive) breast cancer cells MCF-7 and T47D showed significant upregulation compared with the ER-negative BT-549, SK-BR-3 and MDA-MB-231 cells (Figure 1aA). Furthermore, we modeled the development of acquired tamoxifen resistance in patients by treating MCF-7 and T47D cells with 1 *μ*M of 4-hydroxytamoxifen (TAM) for 12 months to select the tamoxifen-resistant cells (named MCF-7/TAM-1, MCF-7/TAM-2, T47D/TAM-1 and T47D/TAM-2). Our results indicated that all these tamoxifen-resistant cells were significantly more resistant (for MCF-7: >20-folds; for T47D: >4-folds) to TAM treatment relative to the parental cells (Supplementary Figure S1). We found that tamoxifen-resistant MCF-7 and T47D cells exhibited lower miR-27b-3p levels compared with the parental cells (Figures 1aB and C).

To further confirm the association between miR-27b-3p and breast cancer, we detected the expression of miR-27b-3p in 19 paired breast cancer and adjacent normal tissues. We found tumor tissue specimens exhibited generally lower miR-27b-3p levels compared with their adjacent normal tissues (Figure 1b). Moreover, miR-27b-3p expression was significantly higher in the tamoxifen-resistant breast cancer tissues from patients with tamoxifen-treated than the tumor tissues from patients who were not treated using any chemotherapy drugs (Figure 1c).

### MiR-27b-3p is repressed by tamoxifen and induced by estrogen in breast cancer cells

**Figure 2:**
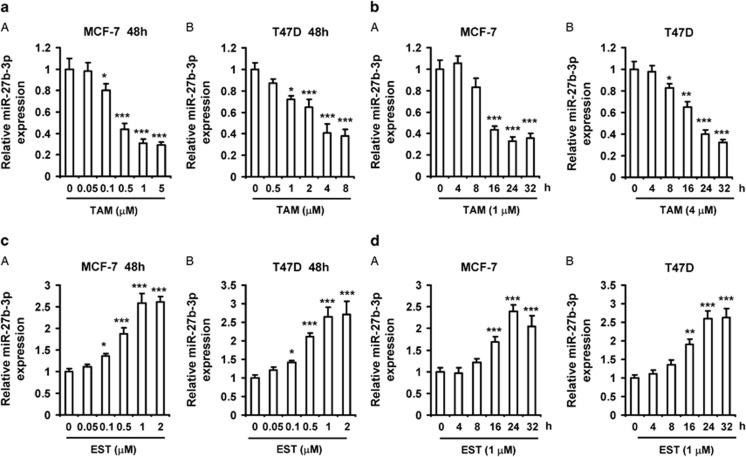
MiR-27b-3p is repressed by tamoxifen and induced by estrogen in breast cancer cells. (**a**) Tamoxifen inhibited miR-27b-3p expression in a dose-dependent manner. MCF-7 and T47D cells were treated with increasing amounts of 4-hydroxytamoxifen (TAM) for 48 h. (**b**) Tamoxifen inhibits miR-27b-3p expression in a time-dependent manner. MCF-7 and T47D cells were treated with 1 and 4 *μ*M of TAM for different time, respectively. (**c**) Estrogen induced miR-27b-3p expression in a dose-dependent manner. MCF-7 and T47D cells were treated with increasing amounts of 17*β*-estradiol (EST) for 48 h. (**d**) Estrogen induced miR-27b-3p expression in a time-dependent manner. MCF-7 and T47D cells were treated with 1 *μ*M of EST for different time, respectively. RNA were then collected and subjected to RT-PCR analysis of gene expression. The levels of miR-27b-3p were presented as fold change compared with the levels in control cells. Columns, means of three determinations; bars, S.D. Results shown are representative of three independent experiments. *P<0.05; **P<0.01; ***P<0.001, compared with DMSO-treated cells

To examine whether tamoxifen regulated miR-27b-3p expression, MCF-7 and T47D cells were treated with increasing doses of TAM for 48 h, and then the levels of miR-27b-3p were determined by RT-PCR. We indicated that miR-27b-3p expression was remarkably inhibited by TAM in a concentration-dependent manner (Figure 2a). Additionally, we detected the levels of miR-27b-3p in both MCF-7 and T47D cells treated with the indicated dose of TAM for different time (2–32 h). We observed that the levels of miR-27b-3p gradually decreased in a time-dependent manner during 4–32 h (Figure 2a). Next, to determine whether estrogen induced miR-27b-3p expression in breast cancer cells, MCF-7 and T47D cells were treated with increasing doses of 17*β*-estradiol (EST) for 48 h, and then the levels of miR-27b-3p were determined by RT-PCR. To avoid the effects of estrogen-derived medium, MCF-7 and T47D cells were cultured in estrogen-free medium for 3 days before incubation with EST. In contrast to TAM, a trend of induction of miR-27b-3p expression by EST in a dose-dependent manner was observed in both breast cancer cells (Figure 2c). Moreover, we also showed EST increased the expression of miR-27b-3p in a time-dependent manner (Figure 2d).

### MiR-27b-3p enhances tamoxifen-induced apoptosis in breast cancer cells

**Figure 3:**
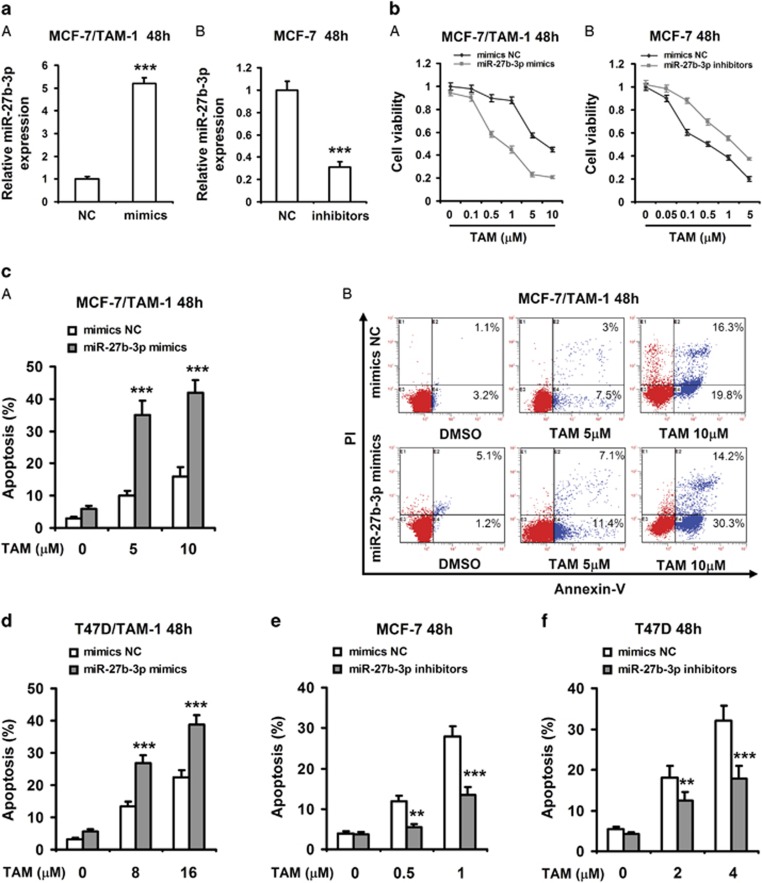
MiR-27b-3p enhances breast cancer cells apoptosis induced by tamoxifen. (**a**) MCF-7/TAM-1 and MCF-7 cells were transfected with miR-27b-3p mimics (**A**) or miR-27b-3p inhibitors (**B**) and the negative control (NC) for 48 h, respectively. RT-PCR was performed to detect the expression of miR-27b-3p. (**b**) MCF-7/TAM-1 and MCF-7 cells were transfected with miR-27b-3p mimics (**A**) or miR-27b-3p inhibitors (**B**) and NC for 8 h, and then cells were treated with the indicated dose of 4-hydroxytamoxifen (TAM) for additional 48 h. MTT assay was performed to examine cell viability. (**c–f**) MCF-7/TAM-1 and T47D/TAM-1 cells were transfected with miR-27b-3p mimics and NC (**c** and **d**); MCF-7 and T47D cells were transfected with miR-27b-3p inhibitors and NC (**e** and **f**). After transfection for 8 h, and then cells were treated with indicated dose of TAM for additional 48 h. Cell apoptosis was assessed by Annexin V-FITC/PI staining assay by flow cytometry. Columns, means of three determinations; bars, S.D. **P <0.01; ***P<0.001, compared with NC-treated cells

To investigate the role of miR-27b-3p in the sensitivity of the breast cancer cells to tamoxifen, breast cancer cells were treated with miR-27b-3p mimics and inhibitors, respectively, and the cytotoxicity induced by TAM was examined. Forced expression of miR-27b-3p mimics in MCF-7/TAM-1 and T47D/TAM-1 cells significantly increased miR-27b-3p levels (Figure 3aA and Supplementary Figure S2Aa). MiR-27b-3p mimics significantly increased the cytotoxicity of TAM in both tamoxifen-resistant breast cancer cells (Figure 3bA and Supplementary Figure S2Ba). On the contrary, miR-27b-3p inhibitors apparently decreased miR-27b-3p levels in MCF-7 and T47D cells (Figure 3aB and Supplementary Figure S2Ab). Moreover, downregulation of miR-27b-3p reduced cell toxicity of TAM in both MCF-7 and T47D cells (Figure 3bB and Supplementary Figure S2Bb).

To further determine the role of miR-27b-3p in increasing the sensitivity of cancer cells to TAM, the mimics and inhibitors-transfected cells were treated with TAM, and apoptosis was detected by annexin V and propidium iodide (PI) staining assay. Our data indicated that miR-27b-3p mimics observably increased TAM-induced cell apoptosis in MCF-7/TAM-1 and T47D/TAM-1 cells (Figures 3c and d). In contrast, inhibition of miR-27b-3p resulted in a significant decrease in the percentages of cell apoptosis stimulated by TAM in MCF-7 and T47D cells (Figures 3e and f).

### Identification of NR5A2 and CREB1 as direct targets of miR-27b

Since miRNAs perform biological functions through negatively regulating their target genes, we predicted the potential targets of miR-27b-3p by using online miRNA target bioinformatics prediction databases (TargetScan, PicTar4, miRDB, miRWalk and miRanda). We initially predicted 126 target genes of miR-27b-3p (Supplementary Table S1). Based on the biological functions of these 126 target genes, we selected NR5A2, CREB1, VAV3, GOLM1, EGFR, FOXO1 and IRS1 as candidate target genes. Subsequently we tested the expression of these seven genes in tamoxifen-sensitive cells MCF-7 and tamoxifen-resistant cells MCF-7/TAM-1 and MCF-7/TAM-2. Results showed NR5A2, CREB1 and GOLM1 expression levels were significantly higher in tamoxifen-resistant breast cancer cells (Supplementary Figure S3). To further validate targeting of NR5A2, CREB1 and GOLM1 by miR-27b-3p, the mRNA levels of all three genes were detected in MCF-7/TAM-1 cells transfected miR-27b-3p mimics and MCF-7 cells transfected miR-27b-3p inhibitors. We found that miR-27b-3p inhibitors significantly increased expression of NR5A2 and CREB1 in MCF-7/TAM-1 cells, whereas miR-27b-3p mimics led to obvious reduction of NR5A2 and CREB1 levels (Supplementary Figures S4A and B). However, there were no significant changes in the mRNA levels of GOLM1 (Supplementary Figures S4A and B).

**Figure 4:**
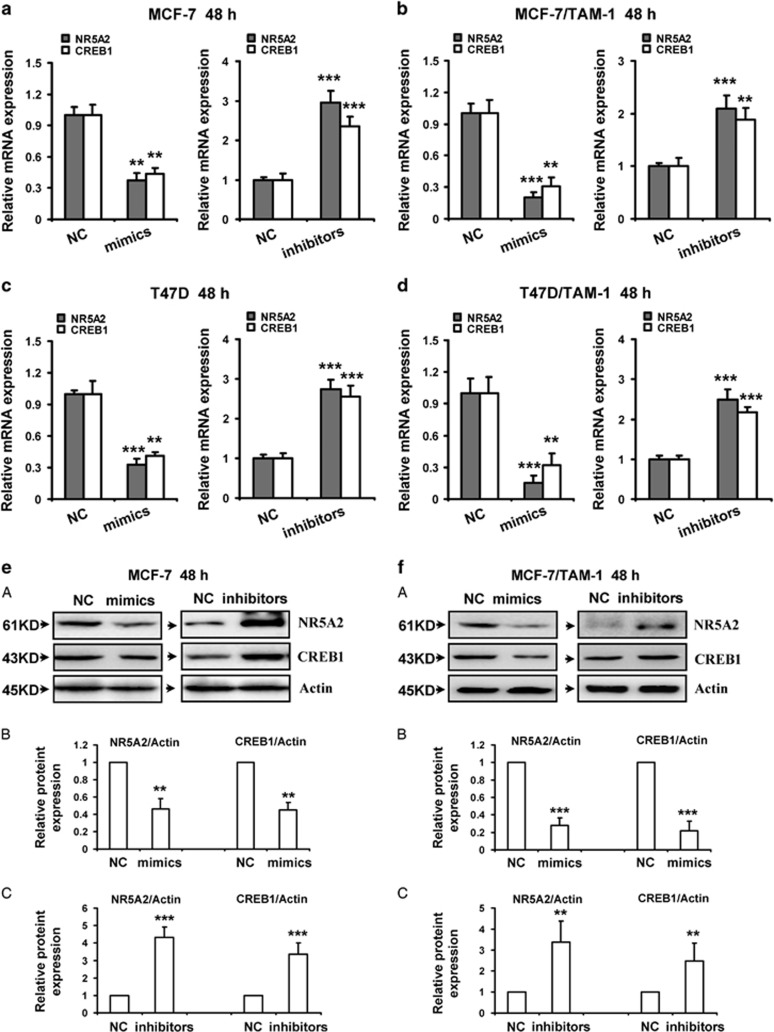

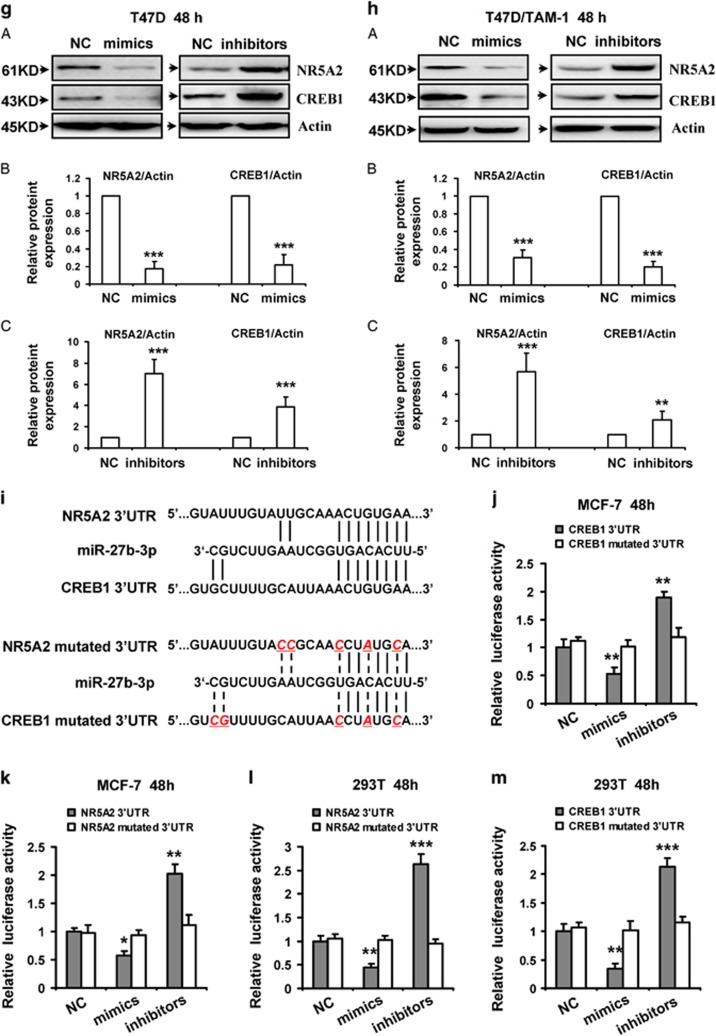
NR5A2 and CREB1 are direct targets of miR-27b-3p. The mRNA expression (**a–d**) and protein levels (**e–h**) of NR5A2 and CREB1 were downregulated by miR-27b-3p in breast cancer cells. MCF-7 (**a** and **e**), MCF-7/TAM-1 (**b** and **f**), T47D (**c** and **g**) and T47D/TAM-1 (**d** and **h**) cells were transfected with miR-27b-3p mimics or inhibitors and the negative control (NC), respectively. RT-PCR was performed to detect the mRNA expression of NR5A2 and CREB1. Western blot was performed to detect the protein expression of NR5A2 and CREB1. Actin was used as a loading control. Data were from three independent experiments. (**e–h**) (**B**) and (**C**) Relative protein levels of NR5A2/Actin and CREB1/Actin were quantified using Image J software. Data are mean±S.D. from three independent experiments. **P<0.01; ***P<0.001, compared with the control group. (**i**) The predicted miR-27b-3p target sites in the 3′UTR of NR5A2 and CREB1 mRNA and their mutated version. (**j–m**) Luciferase activity assays in MCF-7 and 293T cells showed that miR-27b-3p inhibited the expression of NR5A2 and CREB1. MCF-7 and 293T cells were cotransfected with pGL3 vector containing the wild type or mutated 3′UTR of NR5A2 and CREB1, pGL3-Control vector, along with miR-27b-3p mimics and inhibitors or NC. After 48 h, luciferase activity was detected. Data were normalized to luciferase activity in the corresponding cells transfected with NC and are represented as the mean±S.D. of three replicates

Moreover, inhibition of NR5A2 and CREB1 by miR-27b-3p was further validated in MCF-7, T47D, MCF-7/TAM-1 and T47D/TAM-1 cells. Our results showed that miR-27b-3p mimics significantly attenuated both protein and mRNA levels of NR5A2 and CREB1, whereas miR-509-5p inhibitors observably enhanced the protein and mRNA levels of the two genes in all the four breast cancer cells (Figure 4a–h). Additionally, the conserved miR-27b-3p target sites in the 3′-untranslated regions (3′-UTRs) of NR5A2 and CREB1 were predicted, respectively (Figure 4i).

To further confirm targeting of NR5A2 and CREB1 by miR-27b-3p, luciferase activity assay was performed. The wild type (WT) or mutated (MT) 3′-UTRs of NR5A2 and CREB1 were cloned into the downstream of firefly luciferase coding region in pGL3 luciferase reporter vector (Figure 4i). The vectors were cotransfected with miR-27b-3p mimics or inhibitors into 293T and MCF-7 cells. As expected, miR-29b-3p mimics significantly decreased luciferase activity in both 293T and MCF-7 cells transfected WT reporter vectors. However, no obvious reduction of luciferase activities by miR-29b-3p mimics was observed in both 293T and MCF-7 cells transfected with MT reporter vectors (Figures 4j–m). Contrarily, luciferase activities in MCF-7 and 293T cells cotransfected with miR-27b-3p inhibitors and WT reporter vectors were higher than that in cells treated with negative control (NC) miRNA (Figures 4j–m). Additionally, the luciferase activities of the MT reporter vectors were not induced by miR-27b-3p inhibitors in the two cells. Above results implied that miR-27b-3p suppressed the NR5A2 and CREB1 expression by binding 3 ′UTR of the two genes mRNA.

### MiR-27b-3p attenuated breast cancer cell resistance to tamoxifen by repressing NR5A2 and CREB1

Previous study shows that NR5A2 promotes breast cancer cell resistance to tamoxifen involved in inducing ER*α* expression.^20^ Additionally, CREB1 enhances tamoxifen resistance involved in aromatase induction in tamoxifen-resistant breast cancer.^21^ Therefore, we examined whether NR5A2 and CREB1 induced ER and aromatase expression. By western blot and RT-PCR, we showed NR5A2 upregulated ER*α* protein expression and the mRNA levels of ER*α* and ER*β* (Supplementary Figures S5A and C). However, ER*α* and ER*β* expression were not regulated by CREB1 (Supplementary Figure S5D). In addition, we also found overexpression of CREB1 increased aromatase expression (Supplementary Figure S5B).

**Figure 5:**
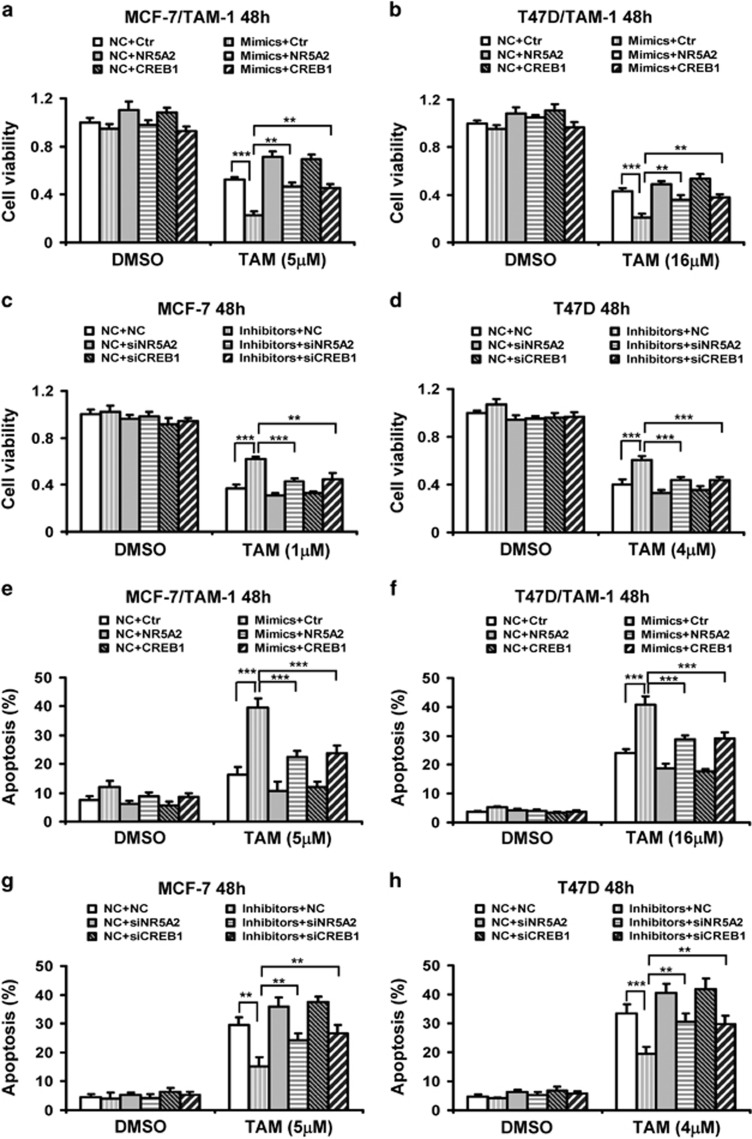
Overexpression of NR5A2 and CREB1 reverses reduction of cell viability and induction of apoptosis by miR-27b-3p mimics, and depletion of NR5A2 and CREB1 reverses induction of cell viability and reduction of apoptosis by miR-509-5p inhibitors in tamoxifen-treated cells. (**a–h**) MCF-7/TAM-1 (**a**) and T47D/TAM-1 (**b**) cells were cotransfected with negative control (NC) or miR-27b-3p mimics along with control (Ctr) or NR5A2 or CREB1 vectors. MCF-7 (**c**) and T47D (**d**) cells were cotransfected with NC or miR-27b-3p inhibitors along with NC or NR5A2 or CREB1 siRNA. After 8 h, cells were treated with indicated dose of 4-hydroxytamoxifen (TAM) for additional 48 h. (**a–d**) MTT assay was performed to examine cell viability. (**e–h**) Cell apoptosis was assessed by Annexin-V-FITC/PI staining assay by flow cytometry. Columns, means of three determinations; bars, S.D.; **P<0.01; ***P<0.001, compared with NC-treated cells

To further determine the functional significance of NR5A2 and CREB1 suppression in miR-27b-3p-mediated tamoxifen sensitivity, MCF-7/TAM-1 and T47D/TAM-1 cells were cotransfected with miR-27b-3p mimics along with NR5A2 or CREB1 overexpression constructs, subsequently cells were treated with TAM. Our results showed TAM-inhibited cell viability was substantially decreased by miR-27b-3p, whereas NR5A2 or CREB1 overexpression significantly reversed the TAM-sensitizing effects of miR-27b-3p in both cells (Figures 5a and b). We showed NR5A2 and CREB1 overexpression vectors distinctly increased the expressions of NR5A2 and CREB1, respectively (Supplementary Figures S6A, C, E and F). Moreover, by Annenxin V/PI staining to quantify the percentage of cells apoptosis, we also found NR5A2 or CREB1 overexpression remarkably attenuated the increase of TAM-induced apoptosis by miR-27b-3p in MCF-7/TAM-1 and T47D/TAM-1 cells (Figures 5e and f).

In addition, MCF-7 and T47D cells were cotransfected with miR-27b-3p inhibitors along with NR5A2 or CREB1 small interfering RNAs (siRNAs), and then treated with TAM. NR5A2 and CREB1 siRNA evidently depleted the expression of NR5A2 and CREB1 in MCF-7 and T47D cells (Supplementary Figures S6B and D). Cell viability assay indicated depletion of NR5A2 or CREB1 obviously reversed the TAM-resistant effects of miR-27b-3p inhibitors in both cell types (Figures 5c and d). Similarly, by detection of cells apoptosis using Annenxin V/PI staining assay, we also found that reduction of TAM-induced apoptosis by miR-27b-3p inhibitors was totally reversed by NR5A2 or CREB1 siRNAs in both MCF-7 and T47D cells (Figures 5g and h). In addition, we also showed that increase of TAM-induced cell death by miR-27b-3p mimics were totally reversed by overexpression of NR5A2 combined with CREB1 in MCF-7/TAM-1 cells (Supplementary Figures S7A and B). All these results suggested that miR-27b-3p attenuated breast cancer cell resistance to TAM by repressing NR5A2 and CREB1.

### MiR-27b-3p enhances sensitivity of breast tumor to tamoxifen in xenograft tumor models

**Figure 6:**
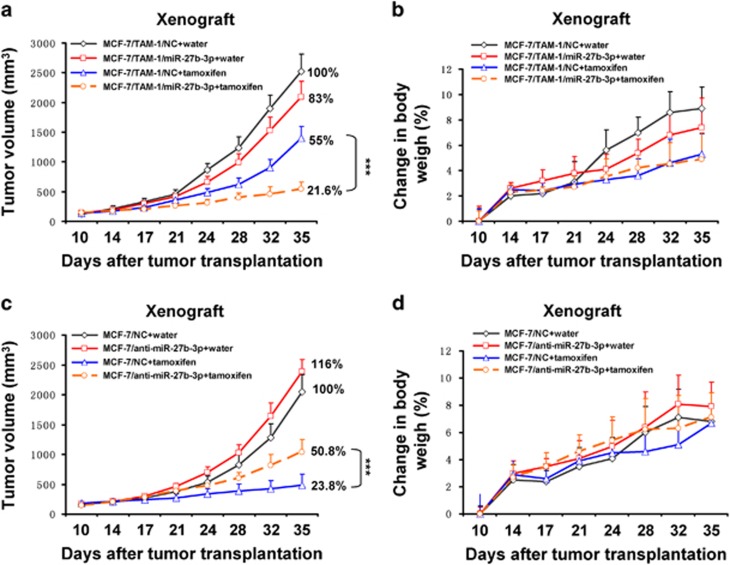
MiR-27b-3p enhances sensitivity of breast tumor to tamoxifen in xenograft tumor models. (**a** and **b**) MCF-7/TAM-1 cells stably expressing miR-27b-3p mimics or control were injected into nude mice. Nude mice were administered with unitary dose of tamoxifen (120 mg/kg per day). (**a**) Tumor volume was measured two times a week by using calipers (as indicated at each time point) for 25 days. (**b**) Average body weight changes were measured over the course of the study. (**c** and **d**) MCF-7 cells stably expressing miR-27b-3p inhibitors (anti-miR-27b-3p) or control were injected into nude mice. Nude mice were administered with a unitary dose of tamoxifen (30 mg/kg per day). (**c**) Tumor volume was measured two times a week by using calipers (as indicated at each time point) for 25 days. (**d**) Average body weight changes were measured over the course of the study. Data are shown as mean±S.D. (*n*=6 per group). ***P<0.001

To determine whether miR-27b-3p enhanced sensitivity of breast tumor to tamoxifen *in vivo*, MCF-7/TAM-1 cells stably expressing miR-27b-3p mimics were injected subcutaneously into the flank of female nude mice. Subsequently the nude mice were administered with tamoxifen, and the volumes of tumors as well as body weight of mice were measured. Results showed that both cell lines stably overexpressing miR-27b-3p exhibited significantly increased sensitivity to tamoxifen (Figure 6a). Notably, no significant differences of body weight in mice were found between the different treatment groups (Figure 6b). Moreover, we evaluated the antitumor efficacy of tamoxifen in mice bearing tumors originating from tamoxifen-sensitive MCF-7 cells stably expressing miR-27b-3p inhibitors (anti-miR-27b-3p). Results showed that tumors stably expressing anti-miR-27b-3p were more resistant to tamoxifen compared with these tumors stably expressing negative control (Figure 6c). Additionally, there were no significant differences of body weight in mice among the four groups (Figure 6d). Above results suggested that miR-27b-3p enhanced sensitivity of breast tumor to tamoxifen in xenograft tumor models.

### MiR-27b-3p is negatively correlated with NR5A2 and CREB1 mRNA levels in breast cancer

**Figure 7:**
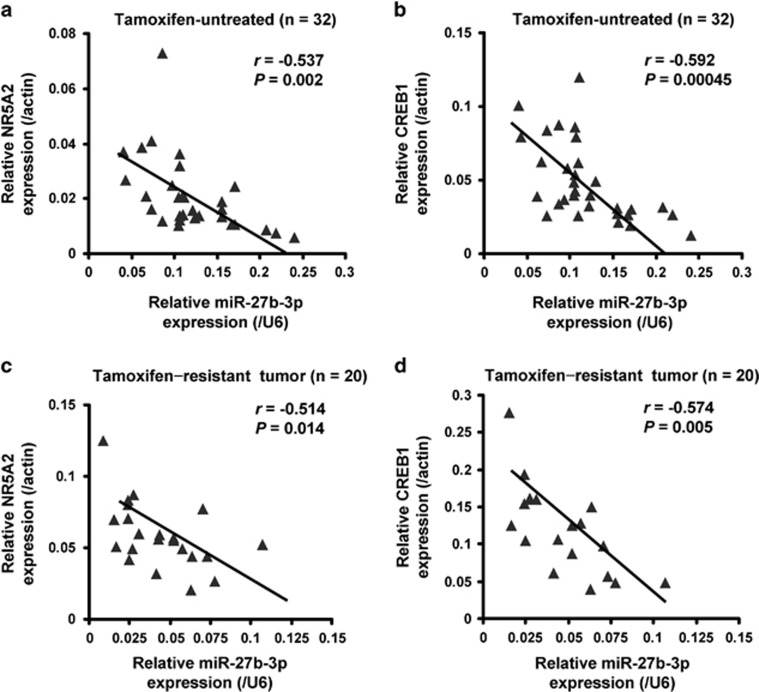
MiR-27b-3p is negatively correlated with NR5A2 and CREB1 mRNA levels in breast cancer. (**a** and **b**) Relative expression of NR5A2 (**a**) and CREB1 (**b**) along with miR-27b-3p was determined by RT-PCR in 32 breast cancer tissues from patients with untreated-tamoxifen. (**c** and **d**) Relative expression of NR5A2 (**c**) and CREB1 (**d**) along with miR-27b-3p was determined by RT-PCR in 20 breast cancer tissues from tamoxifen-resistant patients. For NR5A2 and CREB1, *β*-Actin was used as an internal control; for miR-27b-3p, U6 was used as an internal control. Their expression correlation was analyzed by correlation coefficient and *t*-test

To further examine whether miR-27b-3p levels were correlated with NR5A2 and CREB1, we analyzed NR5A2 and CREB1 mRNA levels in 32 breast cancer tissues from tamoxifen-untreated patients and 20 breast cancer tissues from tamoxifen-resistant patients by RT-PCR. The clinical information of the 52 patients is shown in Supplementary Table S2. By correlation analysis between miR-27b-3p and NR5A2, we found significantly negative correlations between miR-27b-3p and NR5A2 mRNA expression in both untreated-tamoxifen (*r*=−0.537, *P*=0.002; Figure 7a) and tamoxifen-resistant tissue specimens (*r*=−0.514, *P*=0.014; Figure 7c). In addition, correlation analysis was also performed between miR-27b-3p and CREB1 in the two types of tissue specimens. Similarly, significantly negative correlations between miR-27b-3p and CREB1 mRNA expression were found in both untreated-tamoxifen (*r*=−0.592, *P*=0.00045; Figure 7b) and tamoxifen-resistant tissue specimens (*r*=−0.574, *P*=0.005; Figure 7d). These data implied that miR-27b-3p inhibited the expression of NR5A2 and CREB1 *in vivo.*

## Discussion

Although tamoxifen therapy is a very effective treatment for women with ER*α*-positive breast tumors by inhibiting the ER pathway, this therapy exerts very low effects in some patients with *de novo* resistance or acquired resistance. Currently there are only a few useful molecular markers to guide the use of tamoxifen for breast cancer patients with ER positive. Previous studies have revealed important roles of miRNAs in breast cancer resistant to tamoxifen.^22, 23, 24^ Downregulation of miR-375 and miR-873 are associated with tamoxifen resistance, whereas overexpression of miR-375 and miR-873 increases tamoxifen sensitivity in breast cancer.^22, 23^ In this study, we identified miR-27b-3p exhibited decreased expression in tamoxifen-resistant breast cancer tissues and cells. In addition, we had shown for the first time the role of miR-27b-3p in conferring sensitivity of breast cancer to tamoxifen. This hinted that miR-27b-3p could be used as a new potential marker of tamoxifen-resistant breast cancer.

Here, we found breast tumor tissue samples examined expressed lower levels miR-27b-3p than their adjacent normal tissue. A previous study also demonstrates that miR-27b-3p is significantly downregulated in breast cancer.^18^ Notably, the levels of miR-27b-3p were reduced in two triple-negative breast cancer cell lines BT-549 and MDA-MB-231 compared with the ER-positive breast cancer cell lines examined. This result was consistent with recent finding that the expression levels of miR-27b-3p are downregulated in triple-negative breast cancer.^25^ Conversely, several other studies have shown that miR-27b-3p functions as oncogene in breast cancer and upregulated miR-27b-3p levels indicates poor prognosis of triple-negative breast cancer patients.^14, 26, 27^ All these findings and our results suggested that the functions of miR-27b might be dependent on the specific subtype of breast tumor.

In addition, we manifested that the miR-27b-3p is repressed by tamoxifen and induced by estrogen in breast cancer cells. These results suggested that constitutive downregulation of miR-27b-3p exerted in tamoxifen-resistant breast cancer tissues and cell lines were likely initiated by prolonged tamoxifen treatment. However, the mechanism of miR-27b-3p regulated by tamoxifen and estrogen has not yet been clarified. Estrogen can stimulate the transcriptional activity of ER through promoting the recruitment of various receptor-binding coactivators.^28^ Tamoxifen not only prevents the combination of coactivators and ER, but increases the recruitment of corepressors and causes suppression of certain genes expressions.^24^ These findings raised the possibility that the transcriptional level of miR-27b-3p was directly induced by estrogen and inhibited by tamoxifen, respectively. Further work would be required to establish the mechanisms responsible for the regulation of miR-27b-3p by tamoxifen and estrogen.

Previous study has shown that miR-27b-3p is highly expressed in chemoresistant hepatocellular carcinoma cells, although whether these increase in miR-27b-3p levels directly affect the drug sensitivity of hepatocellular carcinoma cells is not determined.^29^ In contrast to this finding, miR-27b-3p has also been reported to sensitize gastric cancer to chemotherapy by directly targeting inhibition of CCNG1, a well-known negative regulator of p53 stability.^30^ Loss of miR-27b-3p provokes the generation of breast cancer stem cells that show docetaxel resistance and high tumorigenicity.^18^ In this study, downregulation of miR-27b-3p was found in the tamoxifen-resistant breast cancer cells and tissues. Furthermore, we demonstrated that the reduction of miR-27b-3p conferred resistance to tamoxifen in breast cancers by increasing the NR5A2 and CREB1 levels. NR5A2, also known as LRH-1, a member of the nuclear receptor subfamily 5 has been implicated in the progression of breast cancer.^31^ Additionally, it has been reported that a high level of CREB1 is observed in breast cancer and associated with disease progression in breast cancer patients.^32, 33^ Our results also showed NR5A2 and CREB1 were highly expressed in the breast cancer tissues and inversely correlated to miR-27b-3p levels.

Recent study shows that NR5A2 increases breast cancer cell growth by directly binding to the promoter of ER*α* gene and inducing its expression.^20^ Interestingly, NR5A2 is transcriptionally regulated by the ER*α* and mediated the mitogenic effects of estrogen.^34^ Moreover, NR5A2 have been implicated in resistance of breast tumor to tamoxifen by promoting the expression of BCL2 and MYC, two important genes involved in inducing cellular proliferation and resisting cell apoptosis.^35, 36^ On the other hand, although the exact mechanisms by which CREB1 confers resistance of breast cancer to tamoxifen have not yet been elucidated, CREB1 transcriptionally activates numerous critical molecules involved in antiapoptosis, such as BRCA1 and BCL2,^37, 38^ and cellular proliferation, such as cyclin A1 and cyclin D1.^38, 39^ Besides, emerging evidence has suggested that CREB1 enhanced aromatase transactivation in breast cancer cells.^23^ Aromatase as a key enzyme for the biosynthesis of estrogens enhances breast cancer cells resistance to tamoxifen.^40^ Indeed, upregulation of aromatase expression is found in tamoxifen-resistant human breast cancer.^23^ Moreover, our results also indicated that forced CREB1 expression increased aromatase expression. Notably, recent evidence suggests that miR-27b-3p enhances cytotoxicity of drugs by targeting inhibition of ENPP1, which induces ABCG2 expression and cell surface localization and increases drugs efflux.^18^ Therefore, we did not exclude the possibility that loss of miR-27b enhances breast cancer cells resistance to tamoxifen involved in downregulation of ABCG2 expression and activity.

**Figure 8:**
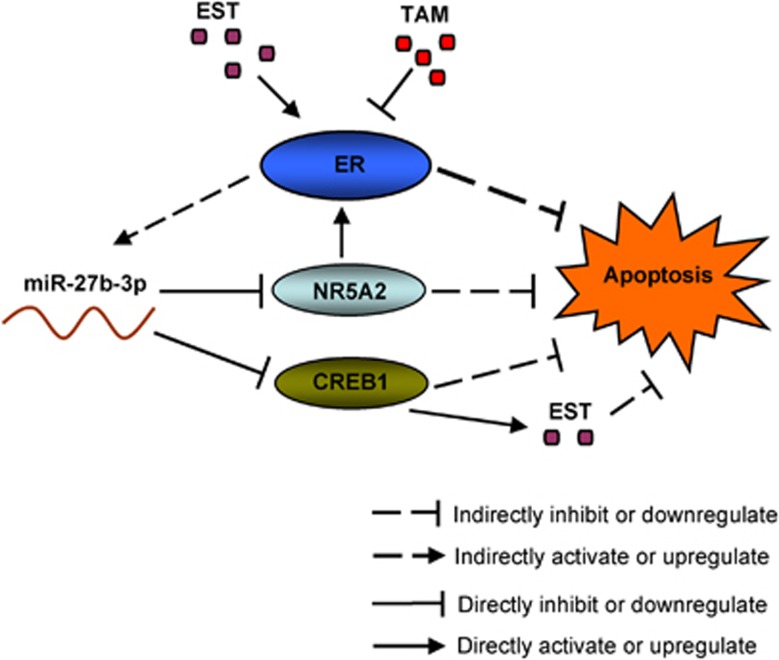
A schematic model depicting miR-27b-3p downregulation promotes tamoxifen resistance in breast cancer cells. Estrogen receptor (ER) inhibitor tamoxifen (TAM) represses miR-27b-3p levels, and estrogen (EST) increases miR-27b-3p levels in breast cancer cells. TAM promotes cell apoptosis by inhibiting ER. On the other hand, miR-27b-3p downregulation by TAM blocks cell apoptosis by upregulating CREB1 and NR5A2. NR5A2 increases ER mRNA levels. CREB1 induces the expression of aromatase, and aromatase promotes the biosynthesis of EST and enhances breast cancer cells resistance to tamoxifen

A genetic regulatory network is depicted in Figure 8 and summarizes the key findings of our study. In conclusion, we had identified miR-27b-3p as a modulating factor for the tamoxifen resistance in breast cancer. Our findings will provide useful information for the development of alternative approaches to diagnose and treat tamoxifen-resistant breast cancer.

## Materials and Methods

### Sample information

ER-positive breast cancer samples were collected from The First Affiliated Hospital of Xi'an Jiaotong University (Xi'an, China), between 2005 and 2009, in which 19 patients contributed paired samples (i.e., both breast tumor samples and adjacent normal tissues were defined as below 1 cm from the tumor tissue) and the other 34 patients contributed breast tumor tissues only. All these samples were neoadjuvant-free and were collected before systemic chemotherapy treatments. In addition, 20 tamoxifen-resistant patients contributed breast tumor tissues after systemic tamoxifen treatments. All of the samples were retrieved within 15 min after the surgery and immediately frozen at −80 °C until used for gene expression analysis as previously described.^41^ This study was approved by the Clinical Ethics Review Board at The First Affiliated Hospital of Xi'an Jiaotong University and written informed consents were from all patients at their recruitment time.

### Cell culture and reagents

Human breast cancer cell line MCF-7 (tamoxifen-sensitive), T47D (tamoxifen-sensitive), BT-549, SK-BR-3, MDA-MB-231 and MCF-10A were purchased from the American Type Culture Collection (ATCC, Manassas, VA, USA). The identities of cell lines were confirmed by using DNA profiling (short tandem repeat, STR). MCF-7/TAM-1 and MCF-7/TAM-2 (tamoxifen-resistant) cell lines were established from MCF-7 cells after the following continuous exposure to 1 *μ*M of TAM (Sigma, St. Louis, MO, USA) for more than 1 year. MCF-10A were cultured in DMEM/F12 (1:1) supplemented with 5% heat-inactivated equine serum, l0 *μ*g/ml insulin 20 ng/ml EGF, 100 ng/ml cholera toxin, 0.5 *μ*g/ml hydrocortisone and 2 mmol/l l-glutamine. BT-549 cells were cultured as previously described.^42^ All other cell lines were maintained in DMEM medium supplemented with 10% fetal bovine serum (Gibco, Carlsbad, CA, USA) at 37 °C in a 5% CO_2_ humidified incubator. Cells were grown in monolayer and passaged routinely 2–3 times a week. Dimethyl sulfoxide (DMSO) and EST was purchased from Sigma.

### Real-time PCR

Total RNA was extracted from breast cancer cell lines and patients tissue using TRIzol reagent (Invitrogen, Carlsbad, CA, USA). Following DNaseI treatment, 2 *μ*g of total RNA was reverse transcribed using cDNA synthesis kit (Bio-Rad, Richmond, CA, USA) to synthesize cDNA specimens. And then, real-time PCR (RT-PCR) analysis of gene expression was performed using 2 *μ*l of cDNA and SYBR Green Supermix (Bio-Rad, Hercules, CA, USA) as recommended by the manufacturer. RT-PCR was conducted by means of the SYBR on the CFX96 system (Bio-Rad). For miRNA, a Ploy-A tail was added to the miRNA, which was then transcribed into cDNA using a universal adaptor primer that included oligo-dT. The generated cDNA was then combined with the Uni-miR RT-PCR Primer (possess the binding site with universal adaptor primer, included in SuperScript III One-Step RT-PCR Kit with Platinum Taq (Invitrogen)) and a miRNA primer (sequence complementary to the miRNA) to complete the RT-PCR reaction. The miRNA expression was normalized using the endogenous U6 snRNA. For the primers, miR-27b-3p: 5′-TTCACAGTGGCTAAGTTCTGC-3′ (forward) and reverse-primer (Uni-miR RT-PCR Primer, included in SYBR Premix Ex Taq kit (TaKaRa, Japan)). U6: 5′-CTCGCTTCGGCAGCACA-3′ (forward) and 5′-AACGCTTCACGAATTTGCGT-3′ (reverse). For mRNA detection, *β*-actin was used as an internal control to normalize gene expression values for each gene expression analysis. For the primers, NR5A2: 5′-GGGTACCATTATGGGCTCCT-3′ (forward) and 5′-TGTCAATTTGGCAGTTCTGG-3′ (reverse);^35^ CREB1: 5′-CCAGCAGAGTGGAGATGCAG-3′ (forward) and 5′-GTTACGGTGGGAGCAGATGAT-3′ (reverse);^43^ others primers are shown in Supplementary Data. The PCR was run in triplicate at 95 °C for 2 min followed by 40 cycles of 95 °C for 15 s, 56 °C for 20 s and 72 °C for 20 s. Comparative quantification was performed using the 2^−^^Δ^^Δ^^Ct^ method as previously described.^44^ Each sample was analyzed in triplicate.

### Plasmids and miRNAs transfection

The NR5A2 and CREB1 expression vectors were purchased from Origene (Rockville, MD, USA). MiR-27b-3p mimics, inhibitors and NC were purchased from GenePharma (Shanghai, China). Cells were trypsinized, counted and seeded into six-well plates the day prior to transfection to ensure 70% cell confluence on the day of transfection. Transfection of miRNA mimics/inhibitors and plasmids into cells was performed using lipofectamine 3000 (Invitrogen) in accordance with the manufacturer's advised procedure as previously described.^45^ The miRNA mimics or inhibitors and plasmids were used at a final concentration of 50 nM and 2 *μ*g, respectively. At 48 h after transfection, RT-PCR and western blotting were performed.

### Determination of cell viability and apoptosis

MTT assay was conducted to assess the cell viability according to the manufacturer's instructions (Sigma). Briefly, cells were plated into 96-well plates at a density of 0.5–1 × 10^4^ cells per well and incubated for at least 8 h in a 5% CO_2_ atmosphere at 37 °C before exposure to vehicle and drugs. The media were then removed, and cells were treated with vehicle and drugs. After the cells were incubated for indicated time, MTT was added to the medium to a final concentration of 0.5 mg/ml. Cells were incubated with MTT for 4 h at 37 °C, and then the medium was removed and 0.2 ml DMSO was added. Absorbance of the media was then measured using a Micro-plate Reader (Bio-Rad) at 570 nm.^46^ This assay was conducted in triplicate.

Measurement of apoptosis was conducted by Annexin V-FITC (fluorescein isothiocyanate)/PI analysis as described previously.^47^ Briefly, cells were seeded and treated with the drugs for 48 h. And then, cells were washed twice with PBS and 1 × 10^6^ cells were resuspended in 1 ml of 1 × Annexin V-binding buffer. Cells undergoing apoptotic cell death were analyzed by counting the cells that stained positive for Annexin V-FITC and negative for PI, and late stage of apoptosis as Annexin V-FITC and PI positive using FACS Calibur flow cytometer (BD Biosciences, San Jose, CA, USA).

### Western blot analysis

Total proteins were isolated from cells with lysis buffer (50 mM Tris, pH 7.5; 150 mM NaCl; 1% NP40; 2.5 mM sodium pyrophosphate; 0.02% sodium azide; 1 mM EGTA, 1 mM EDTA; 1 mM *β*-glycerophosphate; 1 mM Na_3_VO_4_; 1 mM PMSF; 1 *μ*g/ml leupeptin). The lysates were centrifuged at 12 000r.p.m. for 30 min at 4 °C. The protein concentration was determined by Bradford dye method. Equal amounts (20–50 *μ*g) of cell extract were subjected to electrophoresis in 8–12% sodium dodecyl sulfate-polyacrylamide (SDS-PAGE) and transferred to PVDF or nitrocellulose membranes (Millipore, Darmstadt, Germany) for antibody blotting. The membranes were blocked and then incubated with CREB1 antibody (all from Cell Signaling Technologies, Boston, MA, USA), Aromatase, ER*α*, ER*β* and NR5A2 antibody (all from Abcam, Cambridge, UK), *β*-actin antibody (Santa Cruz Biotech, Santa Cruz, CA, USA) overnight at 4 °C. Subsequently, the membranes were incubated with an HRP-conjugated anti-mouse or -rabbit secondary antibody (Protein Tech Group, Chicago, IL, USA) at room temperature for 1 h. The protein bands were visualized using an enhanced chemiluminescence reagent (ECL) kit (GE Healthcare, Munich, Germany), according to the manufacturer's instructions. Blots were quantified using Image J software (National Institutes of Health, Bethesda, MD, USA).^48^

### miRNA target prediction

The analysis of miR-27b-3p predicted targets was performed using the algorithms TargetScan (http://targetscan.org/), PicTar4 (http://pictar.mdc-berlin.de/), miRDB (http://www.mirdb.org/miRDB/), miRWalk (http://zmf.umm.uni-heidelberg.de/apps/zmf/mirwalk/) and miRanda (http://www.microrna.org/microrna).

### Dual-luciferase activity assay

The human 3′-UTR region of NR5A2 and CREB1 genes were amplified by PCR and cloned into the *Xba*I site of the pGL3-Control vector (Promega, Madison, WI, USA), downstream of the luciferase gene, to generate the vector pGL3-NR5A2 and pGL3-CREB1. For luciferase assay, the 293T and MCF-7 cells were cultured in 24-well plates and transfected with 500 ng of pGL3-NR5A2, pGL3-CREB1 or pGL3-control vector along with 50 pmol of miR-27b-3p mimics, inhibitors or NCs, respectively. Transfection of miRNAs was carried out using Lipofectamine 3000 in accordance with the manufacturer's procedure (Life Technologies, Carlsbad, CA, USA). At 24 h after transfection, firefly luciferase activity was measured using the Dual-Luciferase Reporter Assay (Promega) as previously described.^41^ The above experiment was repeated at least three times.

### Animal experiment

Athymic BALB/c nude mice (4–6 weeks old) were obtained from Si-Lai-Ke-Jing-Da Experimental Animal Co. Ltd (Changsha, China). All of the procedures of animal experiments were performed according to approved protocols and in accordance with the guidelines of the Guide for the Care and Use of Laboratory Animals (Institute of Laboratory Animal Resources, Commission on Life Sciences, National Research Council). It was approved by the Institutional Animal Care and Use Committee of Xi'an Jiaotong University (Xi'an, China). MCF-7/TAM-1/NC and MCF-7/TAM-1/mimics cells with vector and stably overexpressed miR-27b-3p were constructed by lentivirus (Genepharma). Cells (4 × 10^6^) were suspended in 0.2 ml of serum-free RPMI/1640 media and were injected into the flanks of female BALB/c nude mice under isoflurane inhalation (4–6 weeks old, *n*=6/group), which were maintained under pathogen-free conditions. One day after tumor cell implantation, mice were treated with tamoxifen and health of mice was checked every day. Unitary dose of tamoxifen was 30 and 120 mg/kg per day in MCF-7 and MCF-7/TAM-1 xenograft mice, respectively. In all cases, water (control) and tamoxifen were administered once a day through oral gavage during 25 days. Tumor volume was measured two times a week by using calipers (as indicated at each time point) for 25 days. The tumor volume was estimated by the following formula: length × width × width × 3.14/6. The mice whole-body weight was measured two times a week as indicated at each time point. All mice were killed by intraperitoneal injection of 200 mg/kg pentobarbital at the end of the experiment.

### Statistical analysis

Results were expressed as mean±S.D. Differences between groups were estimated using the Student's *t*-test. A level of *P*<0.05 was considered to be significant. The relationships between miR-27b-3p and NR5A2 or CREB1 expression level were analyzed by correlation coefficients and linear regression analysis. All analyses were performed using SPSS16.0 software (IBM, Armonk, NY, USA) and a two-tailed value of *P*<0.05 was considered statistically significant.

## Abbreviations

ER: estrogen receptor
TAM: 4-hydroxytamoxifen
miRNAs: microRNAs
NR5A2: nuclear receptor subfamily 5 group A member 2
CREB1: cAMP-response element binding protein 1
siRNA: small interfering RNA

## References

[bib1] Thomas C, Gustafsson JA. The different roles of ER subtypes in cancer biology and therapy. Nat Rev Cancer 2011; 11: 597–608. 21779010 10.1038/nrc3093

[bib2] Lee O, Page K, Ivancic D, Helenowski I, Parini V, Sullivan ME et al. A randomized phase II presurgical trial of transdermal 4-hydroxytamoxifen gel versus oral tamoxifen in women with ductal carcinoma *in situ* of the breast. Clin Cancer Res 2014; 20: 3672–3682. 25028506 10.1158/1078-0432.CCR-13-3045 PMC4101910

[bib3] Davies C, Godwin J, Gray R, Clarke M, Cutter D, Darby S et al. Relevance of breast cancer hormone receptors and other factors to the efficacy of adjuvant tamoxifen: patient-level meta-analysis of randomised trials. Lancet 2011; 378: 771–784. 21802721 10.1016/S0140-6736(11)60993-8 PMC3163848

[bib4] Huang B, Warner M, Gustafsson JA. Estrogen receptors in breast carcinogenesis and endocrine therapy. Mol Cell Endocrinol 2015; 418(Pt 3): 240–244. 25433206 10.1016/j.mce.2014.11.015

[bib5] Jordan VC. The new biology of estrogen-induced apoptosis applied to treat and prevent breast cancer. Endocr Relat Cancer 2015; 22: R1–R31. 25339261 10.1530/ERC-14-0448 PMC4494663

[bib6] Ma N, Zhang W, Qiao C, Luo H, Zhang X, Liu D et al. The tumor suppressive role of MiRNA-509-5p by targeting FOXM1 in non-small cell lung cancer. Cell Physiol Biochem 2016; 38: 1435–1446. 27035590 10.1159/000443086

[bib7] Lan SH, Wu SY, Zuchini R, Lin XZ, Su IJ, Tsai TF et al. Autophagy-preferential degradation of MIR224 participates in hepatocellular carcinoma tumorigenesis. Autophagy 2014; 10: 1687–1689. 25068270 10.4161/auto.29959 PMC4206546

[bib8] Haemmig S, Baumgartner U, Gluck A, Zbinden S, Tschan MP, Kappeler A et al. miR-125b controls apoptosis and temozolomide resistance by targeting TNFAIP3 and NKIRAS2 in glioblastomas. Cell Death Dis 2014; 5: e1279. 24901050 10.1038/cddis.2014.245 PMC4611719

[bib9] Zhang Q, Padi SK, Tindall DJ, Guo B. Polycomb protein EZH2 suppresses apoptosis by silencing the proapoptotic miR-31. Cell Death Dis 2014; 5: e1486. 25341040 10.1038/cddis.2014.454 PMC4237267

[bib10] Zhang Y, Talmon G, Wang J. MicroRNA-587 antagonizes 5-FU-induced apoptosis and confers drug resistance by regulating PPP2R1B expression in colorectal cancer. Cell Death Dis 2015; 6: e1845. 26247730 10.1038/cddis.2015.200 PMC4558495

[bib11] Wei Y, Lai X, Yu S, Chen S, Ma Y, Zhang Y et al. Exosomal miR-221/222 enhances tamoxifen resistance in recipient ER-positive breast cancer cells. Breast Cancer Res Treat 2014; 147: 423–431. 25007959 10.1007/s10549-014-3037-0

[bib12] Cittelly DM, Das PM, Spoelstra NS, Edgerton SM, Richer JK, Thor AD et al. Downregulation of miR-342 is associated with tamoxifen resistant breast tumors. Mol Cancer 2010; 9: 317. 21172025 10.1186/1476-4598-9-317 PMC3024251

[bib13] Ikeda K, Horie-Inoue K, Ueno T, Suzuki T, Sato W, Shigekawa T et al. miR-378a-3p modulates tamoxifen sensitivity in breast cancer MCF-7 cells through targeting GOLT1A. Sci Rep 2015; 5: 13170. 26255816 10.1038/srep13170 PMC4530347

[bib14] Miller TE, Ghoshal K, Ramaswamy B, Roy S, Datta J, Shapiro C et al. MicroRNA-221/222 confers tamoxifen resistance in breast cancer by targeting p27Kip1. J Biol Chem 2008; 44: 29897–29903. 10.1074/jbc.M804612200 PMC2573063 18708351

[bib15] Jin L, Wessely O, Marcusson EG, Ivan C, Calin GA, Alahari SK. Prooncogenic factors miR-23b and miR-27b are regulated by Her2/Neu, EGF, and TNF-alpha in breast cancer. Cancer Res 2013; 73: 2884–2896. 23338610 10.1158/0008-5472.CAN-12-2162 PMC3855090

[bib16] Chen L, Li H, Han L, Zhang K, Wang G, Wang Y et al. Expression and function of miR-27b in human glioma. Oncol Rep 2011; 26: 1617–1621. 21922148 10.3892/or.2011.1458

[bib17] Liu F, Zhang S, Zhao Z, Mao X, Huang J, Wu Z et al. MicroRNA-27b up-regulated by human papillomavirus 16 E7 promotes proliferation and suppresses apoptosis by targeting polo-like kinase2 in cervical cancer. Oncotarget 2016; 7: 19666–19679. 26910911 10.18632/oncotarget.7531 PMC4991410

[bib18] Lee JJ, Drakaki A, Iliopoulos D, Struhl K. MiR-27b targets PPARgamma to inhibit growth, tumor progression and the inflammatory response in neuroblastoma cells. Oncogene 2012; 31: 3818–3825. 22120719 10.1038/onc.2011.543 PMC3290753

[bib19] Takahashi RU, Miyazaki H, Takeshita F, Yamamoto Y, Minoura K, Ono M et al. Loss of microRNA-27b contributes to breast cancer stem cell generation by activating ENPP1. Nat Commun 2015; 6: 7318. 26065921 10.1038/ncomms8318 PMC4490376

[bib20] Thiruchelvam PT, Lai CF, Hua H, Thomas RS, Hurtado A, Hudson W et al. The liver receptor homolog-1 regulates estrogen receptor expression in breast cancer cells. Breast Cancer Res Treat 2011; 127: 385–396. 20607599 10.1007/s10549-010-0994-9

[bib21] Phuong NT, Lim SC, Kim YM, Kang KW. Aromatase induction in tamoxifen-resistant breast cancer: role of phosphoinositide 3-kinase-dependent CREB activation. Cancer Lett 2014; 351: 91–99. 24836190 10.1016/j.canlet.2014.05.003

[bib22] Ward A, Balwierz A, Zhang JD, Kublbeck M, Pawitan Y, Hielscher T et al. Re-expression of microRNA-375 reverses both tamoxifen resistance and accompanying EMT-like properties in breast cancer. Oncogene 2013; 32: 1173–1182. 22508479 10.1038/onc.2012.128

[bib23] Cui J, Yang Y, Li H, Leng Y, Qian K, Huang Q et al. MiR-873 regulates ERalpha transcriptional activity and tamoxifen resistance via targeting CDK3 in breast cancer cells. Oncogene 2015; 34: 4018. 26202215 10.1038/onc.2015.201

[bib24] Musgrove EA, Sutherland RL. Biological determinants of endocrine resistance in breast cancer. Nat Rev Cancer 2009; 9: 631–643. 19701242 10.1038/nrc2713

[bib25] Liu Y, Cai Q, Bao PP, Su Y, Cai H, Wu J et al. Tumor tissue microRNA expression in association with triple-negative breast cancer outcomes. Breast Cancer Res Treat 2015; 152: 183–191. 26062749 10.1007/s10549-015-3460-x PMC4484742

[bib26] Shen S, Sun Q, Liang Z, Cui X, Ren X, Chen H et al. A prognostic model of triple-negative breast cancer based on miR-27b-3p and node status. PLoS One 2014; 9: e100664. 24945253 10.1371/journal.pone.0100664 PMC4063964

[bib27] Wang Y, Rathinam R, Walch A, Alahari SK. ST14 (suppression of tumorigenicity 14) gene is a target for miR-27b, and the inhibitory effect of ST14 on cell growth is independent of miR-27b regulation. J Biol Chem 2009; 284: 23094–23106. 19546220 10.1074/jbc.M109.012617 PMC2755715

[bib28] Shang Y, Hu X, DiRenzo J, Lazar MA, Brown M. Cofactor dynamics and sufficiency in estrogen receptor-regulated transcription. Cell 2000; 103: 843–852. 11136970 10.1016/s0092-8674(00)00188-4

[bib29] Zhuo L, Liu J, Wang B, Gao M, Huang A. Differential miRNA expression profiles in hepatocellular carcinoma cells and drug-resistant sublines. Oncol Rep 2013; 29: 555–562. 23229111 10.3892/or.2012.2155

[bib30] Shang Y, Feng B, Zhou L, Ren G, Zhang Z, Fan X et al. The miR27b-CCNG1-P53-miR-508-5p axis regulates multidrug resistance of gastric cancer. Oncotarget 2016; 7: 538–549. 26623719 10.18632/oncotarget.6374 PMC4808016

[bib31] Lai CF, Flach KD, Alexi X, Fox SP, Ottaviani S, Thiruchelvam PT et al. Co-regulated gene expression by oestrogen receptor alpha and liver receptor homolog-1 is a feature of the oestrogen response in breast cancer cells. Nucleic Acids Res 2013; 41: 10228–10240. 24049078 10.1093/nar/gkt827 PMC3905875

[bib32] Xiao X, Li BX, Mitton B, Ikeda A, Sakamoto KM. Targeting CREB for cancer therapy: friend or foe. Curr Cancer Drug Targets 2010; 10: 384–391. 20370681 10.2174/156800910791208535 PMC4206256

[bib33] Chhabra A, Fernando H, Watkins G, Mansel RE, Jiang WG. Expression of transcription factor CREB1 in human breast cancer and its correlation with prognosis. Oncol Rep 2007; 18: 953–958. 17786359

[bib34] Annicotte JS, Chavey C, Servant N, Teyssier J, Bardin A, Licznar A et al. The nuclear receptor liver receptor homolog-1 is an estrogen receptor target gene. Oncogene 2005; 24: 8167–8175. 16091743 10.1038/sj.onc.1208950 PMC2259230

[bib35] Bianco S, Brunelle M, Jangal M, Magnani L, Gevry N. LRH-1 governs vital transcriptional programs in endocrine-sensitive and -resistant breast cancer cells. Cancer Res 2014; 74: 2015–2025. 24520076 10.1158/0008-5472.CAN-13-2351

[bib36] Serasinghe MN, Missert DJ, Asciolla JJ, Podgrabinska S, Wieder SY, Izadmehr S et al. Anti-apoptotic BCL-2 proteins govern cellular outcome following B-RAF(V600E) inhibition and can be targeted to reduce resistance. Oncogene 2015; 34: 857–867. 24608435 10.1038/onc.2014.21 PMC4160434

[bib37] Mueller CR, Roskelley CD. Regulation of BRCA1 expression and its relationship to sporadic breast cancer. Breast Cancer Res 2003; 5: 45–52. 12559046 10.1186/bcr557 PMC154136

[bib38] Shankar DB, Cheng JC, Kinjo K, Federman N, Moore TB, Gill A et al. The role of CREB as a proto-oncogene in hematopoiesis and in acute myeloid leukemia. Cancer Cell 2005; 7: 351–362. 15837624 10.1016/j.ccr.2005.02.018

[bib39] Kumar AP, Bhaskaran S, Ganapathy M, Crosby K, Davis MD, Kochunov P et al. Akt/cAMP-responsive element binding protein/cyclin D1 network: a novel target for prostate cancer inhibition in transgenic adenocarcinoma of mouse prostate model mediated by Nexrutine, a Phellodendron amurense bark extract. Clin Cancer Res 2007; 13: 2784–2794. 17473212 10.1158/1078-0432.CCR-06-2974 PMC1948816

[bib40] Simpson ER, Mahendroo MS, Means GD, Kilgore MW, Hinshelwood MM, Graham-Lorence S et al. Aromatase cytochrome P450, the enzyme responsible for estrogen biosynthesis. Endocr Rev 1994; 15: 342–355. 8076586 10.1210/edrv-15-3-342

[bib41] Luo X, Yao J, Nie P, Yang Z, Feng H, Chen P et al. FOXM1 promotes invasion and migration of colorectal cancer cells partially dependent on HSPA5 transactivation. Oncotarget 2016; 7: 26480–26495. 27034162 10.18632/oncotarget.8419 PMC5041994

[bib42] Zou Z, Yuan Z, Zhang Q, Long Z, Chen J, Tang Z et al. Aurora kinase A inhibition-induced autophagy triggers drug resistance in breast cancer cells. Autophagy 2012; 8: 1798–1810. 23026799 10.4161/auto.22110 PMC3541289

[bib43] Webb R, Wren JD, Jeffries M, Kelly JA, Kaufman KM, Tang Y et al. Variants within MECP2, a key transcription regulator, are associated with increased susceptibility to lupus and differential gene expression in patients with systemic lupus erythematosus. Arthritis Rheum 2009; 60: 1076–1084. 19333917 10.1002/art.24360 PMC2734382

[bib44] Mi YJ, Geng GJ, Zou ZZ, Gao J, Luo XY, Liu Y et al. Dihydroartemisinin inhibits glucose uptake and cooperates with glycolysis inhibitor to induce apoptosis in non-small cell lung carcinoma cells. PLoS One 2015; 10: e120426. 10.1371/journal.pone.0120426 PMC4370589 25799586

[bib45] Lou YF, Zou ZZ, Chen PJ, Huang GB, Li B, Zheng DQ et al. Combination of gefitinib and DNA methylation inhibitor decitabine exerts synergistic anti-cancer activity in colon cancer cells. PLoS One 2014; 9: e97719. 24874286 10.1371/journal.pone.0097719 PMC4038521

[bib46] Jiang S, Zou Z, Nie P, Wen R, Xiao Y, Tang J. Synergistic effects between mTOR complex 1/2 and glycolysis inhibitors in non-small-cell lung carcinoma cells. PLoS One 2015; 10: e132880. 10.1371/journal.pone.0132880 PMC4503566 26176608

[bib47] Zou ZZ, Nie PP, Li YW, Hou BX, Rui-Li, Shi XP et al. Synergistic induction of apoptosis by salinomycin and gefitinib through lysosomal and mitochondrial dependent pathway overcomes gefitinib resistance in colorectal cancer. Oncotarget 2015 (e-pub ahead of print 7 Oct 2015; doi:doi:10.18632/oncotarget.5628). 10.18632/oncotarget.5628 PMC5410233 26461472

[bib48] Nie P, Hu W, Zhang T, Yang Y, Hou B, Zou Z. Synergistic induction of erlotinib-mediated apoptosis by resveratrol in human non-small-cell lung cancer cells by down-regulating Survivin and up-regulating PUMA. Cell Physiol Biochem 2015; 35: 2255–2271. 25895606 10.1159/000374030

