# Supplementary material for: Downregulation of microRNA-27b-3p enhances tamoxifen resistance in breast cancer by increasing NR5A2 and CREB1 expression
Source: Cell Death Dis. 2016 Nov 3;7(11):e2454–. doi: 10.1038/cddis.2016.361 (PMC5260890; doi:10.1038/cddis.2016.361)
Supplement: Supplementary Information [file cddis2016361x1.doc]

**Supplementary Materials and Methods**

**Primers for Real-time PCR**

VAV3: 5'-CTGGTGAACAAGGGACACTC-3' (forward) and 5'-TTT AGGAGTTCTTCGCAGTCCATT-3' (reverse); GOLM1: forward 5'-ACCAAAAAGGGGAATGAAGC-3' (forward) and 5'-GTGTCTGGGACTTGCTGTTACC-3' (reverse); EGFR: 5'-ATGCCCGCATTAGCTCTTAG-3' (forward) and 5'-GCAACTTCCCAAAATGTGCC-3' (reverse); FOXO1: 5’-TGGACATGCTCAGCAGACATC-3’ (forward) and 5’-TTGGGTCAGGCGGTTCA-3’ (reverse); IRS1: 5’-CTCTACACCCGAGACGAACAC-3’ (forward) and 5’-TGGGCCTTTGCCCGATTATG-3’ (reverse); β-actin: 5’-TTCTACAATGAGCTGCGTGTG-3’ (forward) and 5’-GGGGTGTTGAAGGTCTCAAA-3’ (reverse). ERα: 5’-GAAAGGTGGGATACGAAAAGACC-3’ (forward) and 5’-GCTGTTCTTCTTAGAGCGTTTGA-3’ (reverse); ERβ: 5’-AGCACGGCTCCATATACATACC-3’ (forward) and 5’-TGGACCACTAAAGGAGAAAGGT-3’ (reverse);

**Supplementary** **figure legend**

**Figure S1** Antiproliferative effects of tamoxifen on sensitive and resistant breast cancer cell lines. (**A** and **B**) (**a**) MCF-7 and T47D as well as tamoxifen-resistant breast cancer cells MCF-7/TAM1, MCF-7/TAM2, T47D/TAM1 and T47D/TAM2 cells were treated with indicated dose of 4-hydroxytamoxifen (TAM) for 48 h. MTT assay was performed to examine cell viability. (**b**) The IC_50_ values were calculated according to the dose-response curves. Means ± SD of three independent experiments performed in triplicate are shown.

**Figure S2** MiR-27b-3p enhances sensitivity of breast cancer cells to tamoxifen. (**A**) T47D/TAM-1 and T47D cells were transfected with miR-27b-3p mimics (**a**) or miR-27b-3p inhibitors (**b**) and the negative control (NC) for 48 h, respectively. RT-PCR was performed to detect the expression of miR-27b-3p. (**B**) T47D/TAM-1 and T47D cells were transfected with miR-27b-3p mimics (**a**) or miR-27b-3p inhibitors (**b**) and NC for 8 h, and then cells were treated with indicated dose of 4-hydroxytamoxifen (TAM) for additional 48 h. MTT assay was performed to examine cell viability. Mean ± SD of three independent experiments performed in triplicate are shown; **, *P* < 0.01; ***, *P* < 0.001, compared with NC-treated cells.

**Figure S3** RT-PCR was performed to detect the mRNA expression of NR5A2, CREB1, VAV3, GOLM1, EGFR, FOXO1 and IRS1 in MCF-7, MCF-7/TAM1 and MCF-7/TAM2 cells. β-Actin was used as an internal control; Mean ± SD of three independent experiments performed in triplicate are shown; **, *P* < 0.01; ***, *P* < 0.001, compared with MCF7 cells.

**Figure S4** MiR-27b-3p decreases the mRNA levels of NR5A2 and CREB1 in breast cancer cells. MCF-7 (**A**) and MCF-7/TAM-1 (**B**) cells were transfected with miR-27b-3p (miR-27b) inhibitors or mimics and the negative control (NC), respectively. RT-PCR was performed to detect the mRNA expression of NR5A2, CREB1 and GOLM1. β-Actin was used as an internal control. Mean ± SD of three independent experiments performed in triplicate are shown; ***, *P* < 0.001, compared with NC-treated cells.

**Figure S5** Overexpression of NR5A2 and CREB1 induces ER and aromatase expression in MCF-7 cells respectively. (**A**-**D**) MCF-7 cells were transfected with control (Ctr) or NR5A2 or CREB1 vectors. After 48 h, Western blot was performed to detect the indicated protein expression (**A** and **B**). RT-PCR was performed to detect the ERα and ERβ mRNA expression (**C** and **D**). β-actin (Actin) was used as a loading control. Data were from three independent experiments. Columns, means of three determinations; bars, SD; ***, *P* < 0.001, compared with control (CTR) vectors treated cells.

**Figure S6** Overexpression of NR5A2 or CREB1 reverses mRNA reduction of NR5A2 and CREB1 by miR-27b-3p mimics, and depletion of NR5A2 or CREB1 reverses mRNA induction of NR5A2 and CREB1 by miR-509-5p inhibitors in breast cancer cells. (**A-D**) MCF-7/TAM-1 (**A**) and T47D/TAM-1 (**C**) cells were cotransfected with nagative control (NC) or miR-27b-3p mimics along with control (Ctr) or NR5A2 or CREB1 vectors. MCF-7 (**B**) and T47D (**D**) cells were cotransfected with NC or miR-27b-3p inhibitors along with NC or NR5A2 or CREB1 siRNA. After 48 h, RT-PCR was performed to detect the mRNA expression of NR5A2 and CREB1. Columns, means of three determinations; bars, SD. **, *P* < 0.01; ***, *P* < 0.001, compared with control cells. (**E** and **F**) MCF-7/TAM-1 cells were cotransfected with NC or miR-27b-3p mimics along with Ctr or NR5A2 or CREB1 vectors. Western blot was performed to detect the protein expression of NR5A2 and CREB1. β-actin (Actin) was used as a loading control. Data were from three independent experiments.

**Figure S7** Overexpression of NR5A2 and CREB1 reverses reduction of cell viability and induction of apoptosis by miR-27b-3p mimics in tamoxifen-treated cells. (**A** and **B**) MCF-7/TAM-1 cells were cotransfected with nagative control (NC) or miR-27b-3p mimics along with control (Ctr) or NR5A2 or CREB1 or NR5A2 combined with CREB1 vectors. After 8 h, cells were treated with indicated dose of 4-hydroxytamoxifen (TAM) for additional 48 h. (**A**) MTT assay was performed to examine cell viability. (**B**) Cell apoptosis was assessed by Annexin-V-FITC/PI staining assay by flow cytometry. Columns, means of three determinations; bars, SD; ***, *P* < 0.001, compared with NC plus NC-treated cells.

**Table S1** The potential targets of miR-27b-3p by using online miRNA target bioinformatics prediction databases (TargetScan, PicTar4, miRDB, miRWalk and miRanda).

| CDH5 | HORMAD2 | DNAJC13 | GRB2 | FLJ10404 | YWHAB | FOXP2 |
| --- | --- | --- | --- | --- | --- | --- |
| CDH11 | C5orf41 | ZDHHC17 | USP25 | PPP1CC | C6orf60 | CD28 |
| PPIF | DCX | MMD | SEC61A1 | FBXW7 | C1orf108 | NPEPPS |
| RCAN2 | MIER3 | **NR5A2** | HOXA10 | NXT2 | NARG1 | BAG2 |
| SPRY2 | DCP2 | ST6GALNAC3 | HOXA13 | C8orf4 | EDEM3 | GFPT2 |
| CDS1 | ZNF800 | MAP3K7IP3 | C2orf55 | PTGER4 | PDHX | NR1D2 |
| **VAV3** | HBEGF | ATXN10 | **IRS1** | SEMA6A | ING5 |  |
| ARFGEF1 | ABCA1 | SGMS1 | LIMK1 | GPAM | DOT1L |  |
| PLK2 | **EGFR** | ZZZ3 | KITLG | RGS1 | BRSK1 |  |
| TLK2 | EN2 | SS18L1 | NDUFS4 | NEUROD6 | LCOR |  |
| RNF139 | TAPT1 | C10orf137 | NEDD4 | MAP2K4 | KIAA1737 | |
| SLITRK1 | NRK | WSB1 | NFE2L2 | SFRP1 | CBFB |  |
| COL19A1 | NR2F6 | NGFRAP1 | NGFR | RMND5A | B4GALT3 | |
| ANKRD43 | C10orf56 | PDE7B | NOVA1 | SLC6A1 | RNGTT |  |
| NCOA7 | C7orf41 | DKK2 | ITSN2 | SMARCA1 | STBD1 |  |
| ADORA2B | PDS5B | STK39 | RBJ | SNAP25 | FAM105B |  |
| **CREB1** | **FOXO1** | GNS | **GOLM1** | TROVE2 | LONRF1 | |
| HAPLN1 | PLCL2 | AQP11 | UBR5 | TEAD1 | RPS6KA5 | |
| UBE2F | CAMTA1 | ANK1 | GALNT7 | UBE2N | MED14 |  |
| C1orf52 | SATB2 | ANK2 | PHB | VIP | SOCS6 |  |

**Table S2 Clinical information of the 52 patients contributed samples** in this study

| **Characteristics** | **Patients with untreated tamoxifen** | | **Patients with tamoxifen resistance** | | |
| --- | --- | --- | --- | --- | --- |
|  | **Number (%)** | | | **Number (%)** | |
| **Age 55 (30-75) years**  <35  35-55  >55  **Histology**  Infiltrating ductal carcinoma  Infiltrating (mixed) carcinoma  Others  **TNM stage**  II  IIIA  IIIB-IV  **Estrogen receptor status**  Negative  Positive  **Progesterone receptor status**  Negative  Positive  **Her2 status**  Negative  Positive | **32**  **5**  **20**  **7**  **15**  **7**  **10**  **5**  **16**  **11**  **0**  **32**  **13**  **19**  **17**  **15** | **15.6**  **62.5**  **21.9**  **46.8**  **21.9**  **31.3**  **15.6**  **50**  **34.4**  **0**  **100**  **40.6**  **59.4**  **53.1**  **46.9** | **20**  **4**  **8**  **8**  **11**  **5**  **4**  **3**  **6**  **11**  **0**  **20**  **8**  **12**  **5**  **15** | | **20**  **40**  **40**  **55**  **25**  **20**  **15**  **30**  **55**  **0**  **100**  **40**  **60**  **25**  **75** |
