# Supplementary figures and images for: Downregulation of microRNA-27b-3p enhances tamoxifen resistance in breast cancer by increasing NR5A2 and CREB1 expression
Source: Cell Death Dis. 2016 Nov 3;7(11):e2454–. doi: 10.1038/cddis.2016.361 (PMC5260890; doi:10.1038/cddis.2016.361)

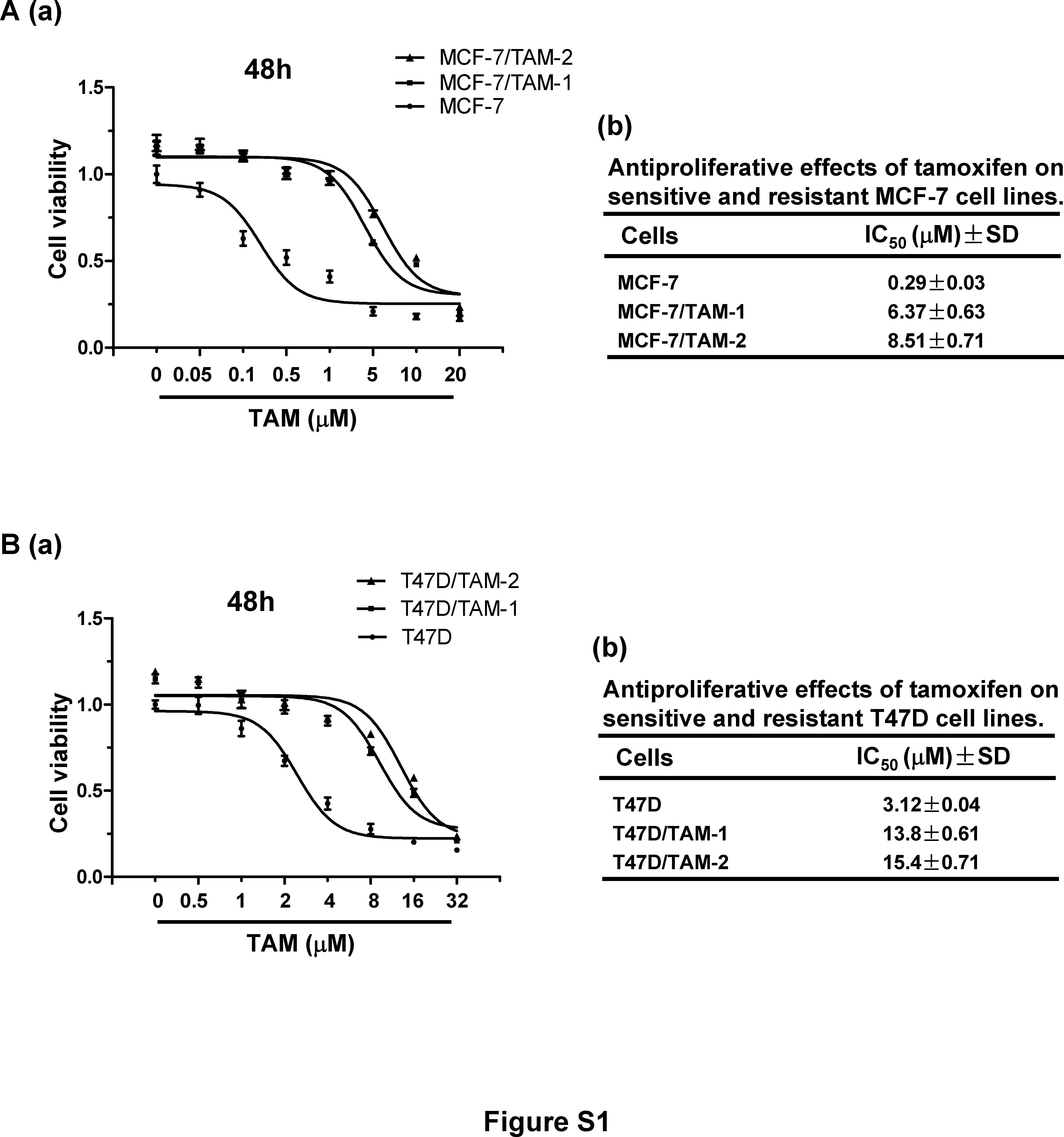

Supplement: Supplementary Figure S1 [file cddis2016361x2.tif]

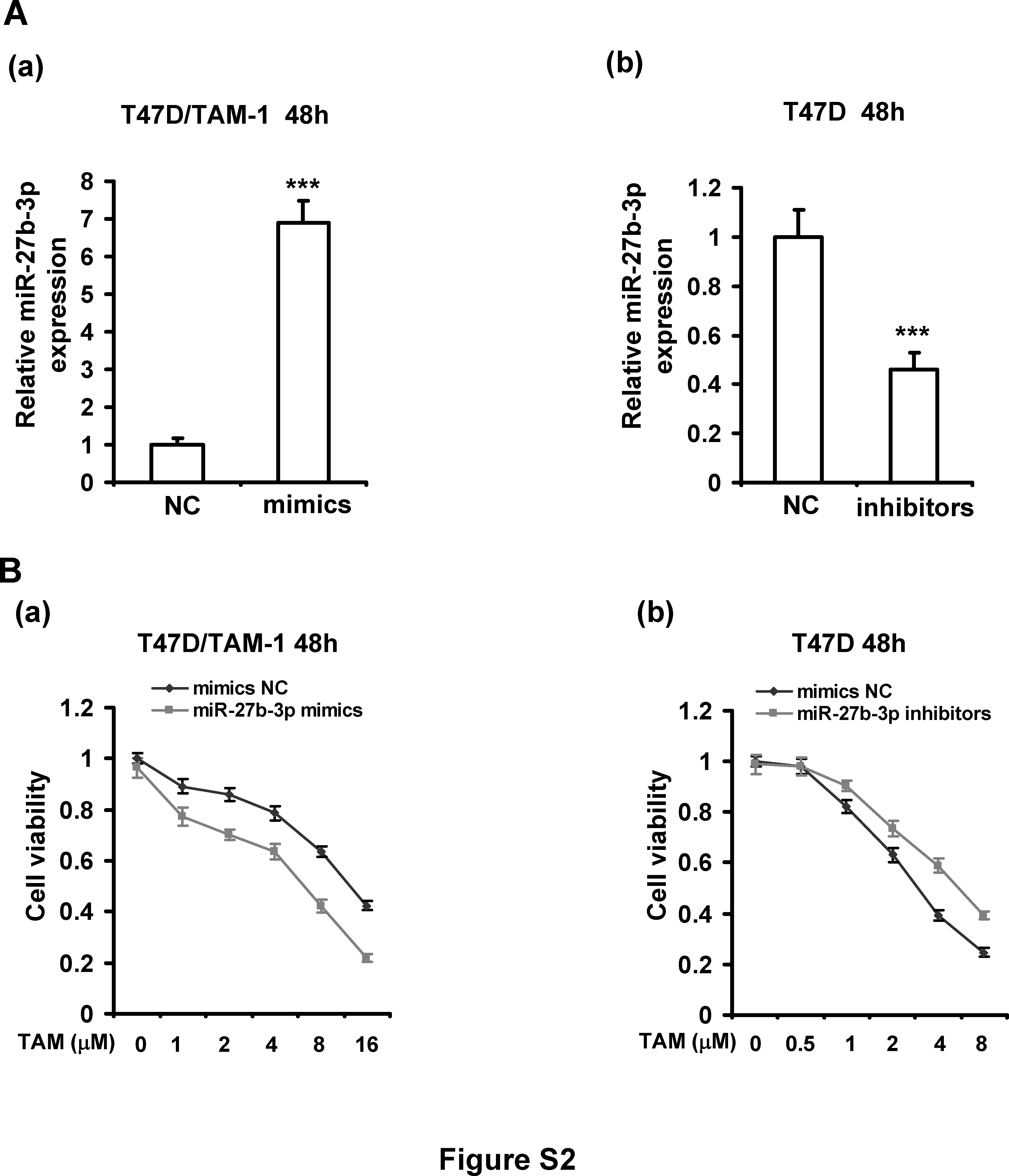

Supplement: Supplementary Figure S2 [file cddis2016361x3.tif]

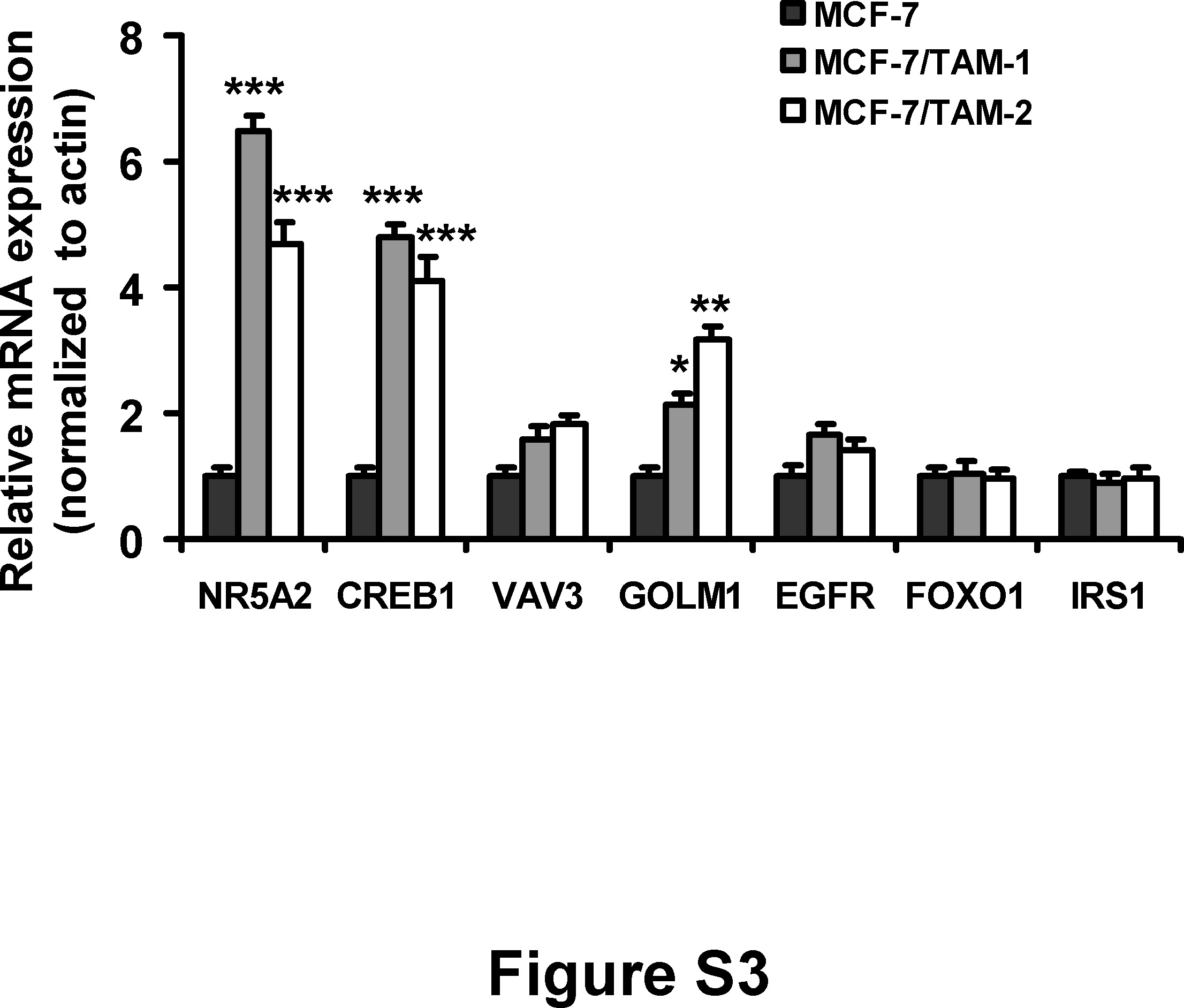

Supplement: Supplementary Figure S3 [file cddis2016361x4.tif]

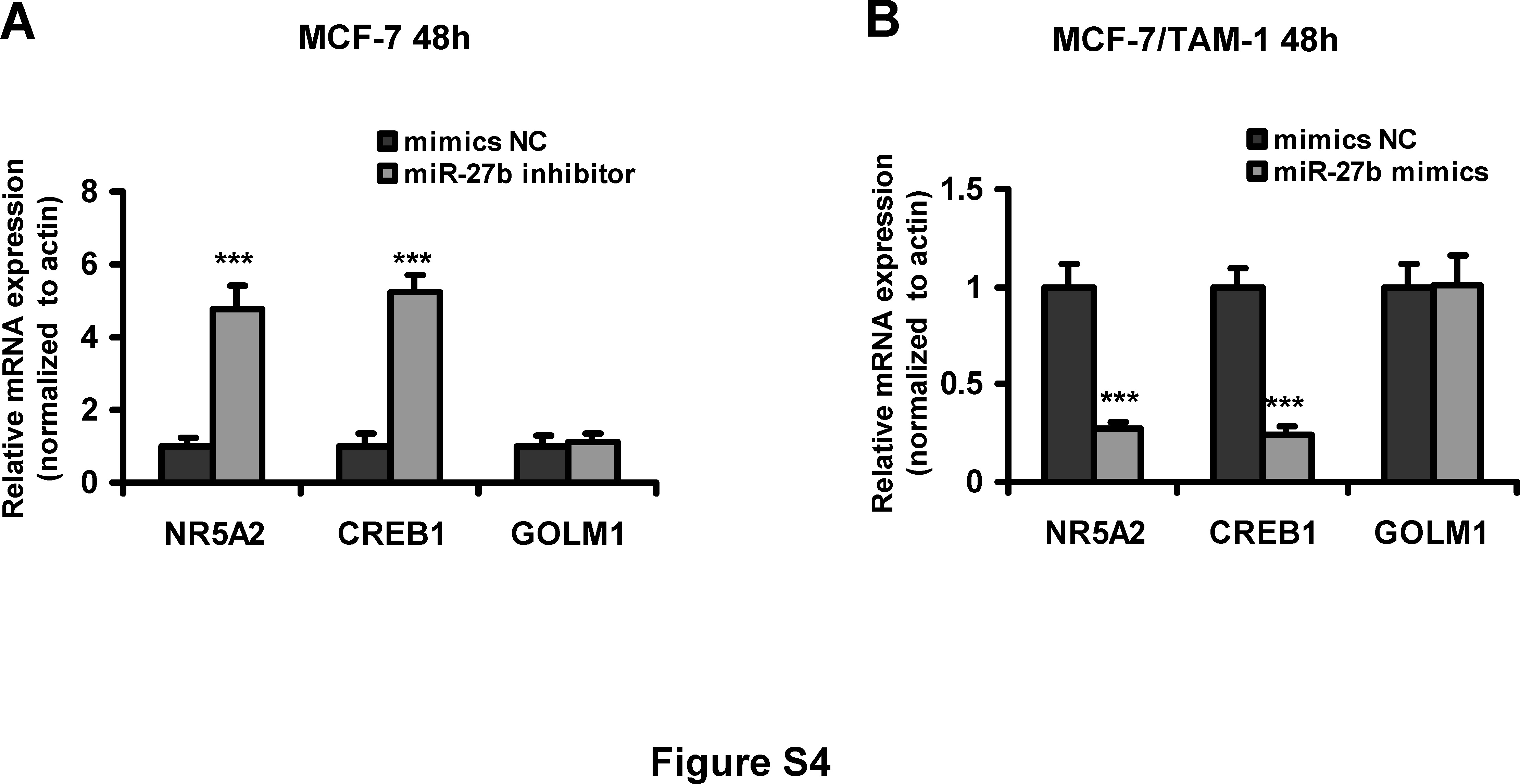

Supplement: Supplementary Figure S4 [file cddis2016361x5.tif]

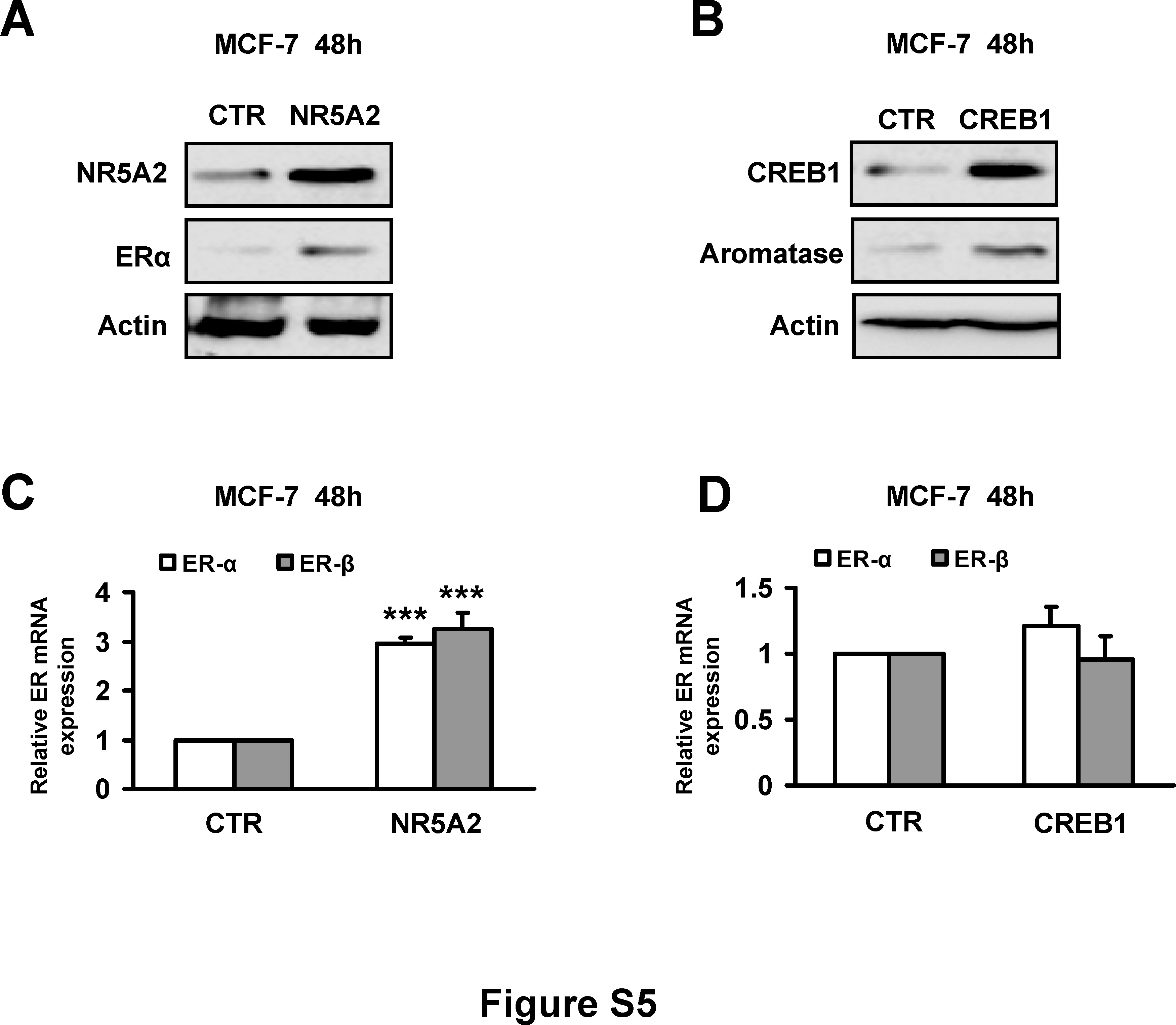

Supplement: Supplementary Figure S5 [file cddis2016361x6.tif]

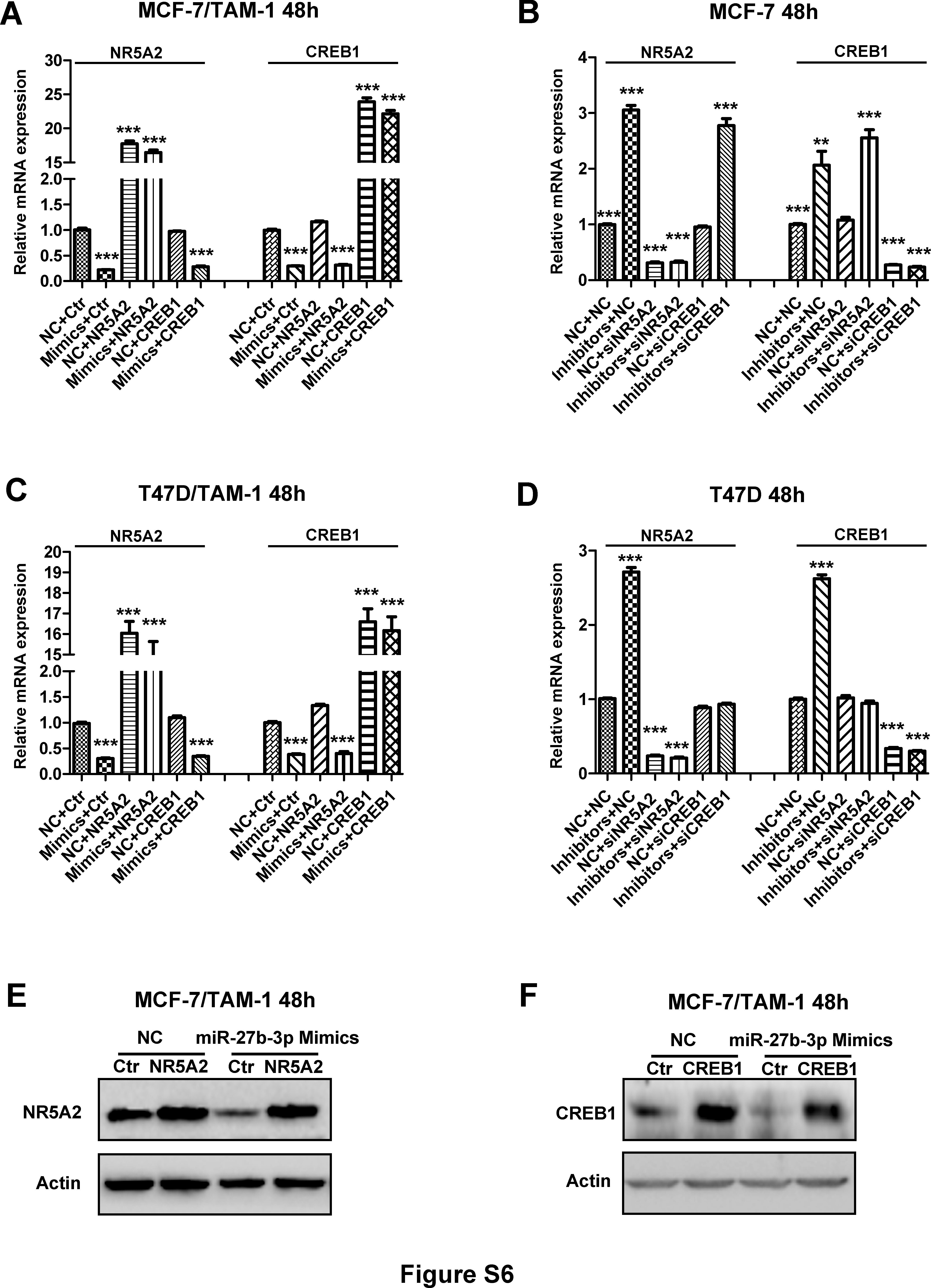

Supplement: Supplementary Figure S6 [file cddis2016361x7.tif]

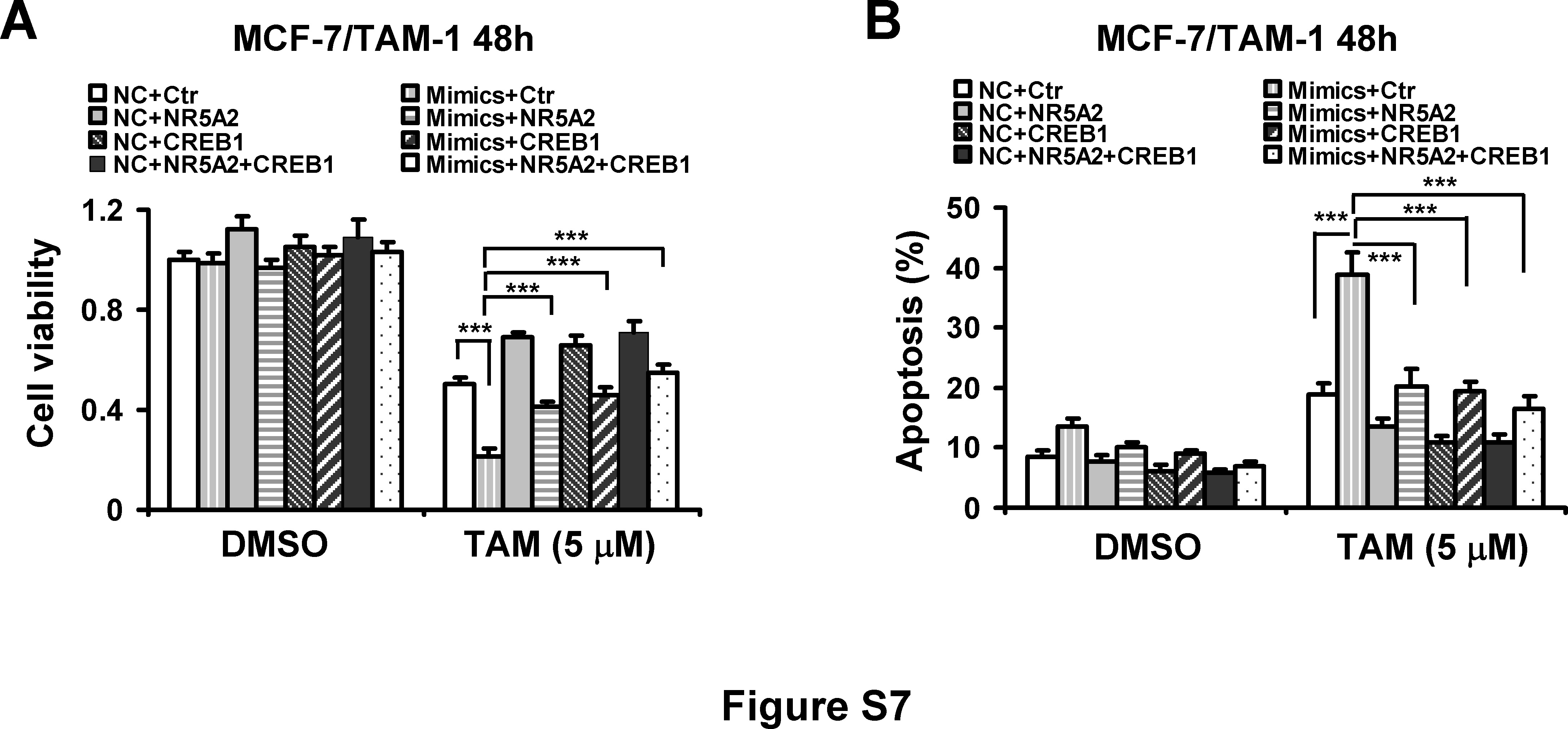

Supplement: Supplementary Figure S7 [file cddis2016361x8.tif]
